# The mental cost of urban living: neighbourhood deprivation, income, and depressive symptoms in Germany

**DOI:** 10.1186/s12889-026-30253-8

**Published:** 2026-11-14

**Authors:** Arieja Farugie, Ana N. Tibubos, Caroline Cohrdes, Tobias Rüttenauer

**Affiliations:** 1 Institute of Psychology, Department of Personality and Psychological Diagnostic, Goethe University Frankfurt, Frankfurt, Germany; 2 Department of Nursing Science, Diagnostics in Healthcare & e- Health, Trier University, Trier, Germany; 3 Department of Psychosomatic Medicine and Psychotherapy, University Medical Center of the Johannes Gutenberg-University Mainz, Mainz, Germany; 4 Department of Epidemiology and Health Monitoring, Robert Koch Institute, Berlin, Germany; 5 Center for Critical Computational Studies (C3S), Goethe University Frankfurt, Frankfurt, Germany

**Keywords:** Depressive symptoms, Neighbourhood deprivation, Socioeconomic inequality, Urban–rural differences, Mental health disparities, Person–environment interaction

## Abstract

**Objective:**

This study investigates the established associations between neighbourhood socioeconomic deprivation, household income, and depressive symptoms in Germany, and assesses whether these associations differ across settlement structures. Building on the person-in-environment framework, we understand mental health as an emergent outcome of the interplay between individuals and their environments.

**Method:**

We analysed cross-sectional data from the nationally representative German Health Update 2019/2020 wave of the European Health Interview Survey (GEDA 2019/2020-EHIS) ​ (*N* = 20,773). Depressive symptoms were measured using Patient Health Questionnaire-8 (PHQ-8). Polynomial regression models tested linear and quadratic associations of neighbourhood deprivation (German Index of Socioeconomic Deprivation, GISD) with depressive symptoms, and their interaction with household income quintiles. Whether settlement type (cities, urban, rural and sparsely populated counties) moderated these associations was tested explicitly using nested interaction models.

**Results:**

Higher neighbourhood deprivation was associated with increased depressive symptoms, with a significant quadratic term indicating disproportionately higher symptom severity in highly deprived areas. Household income was inversely associated with depressive symptoms, though its moderation of the deprivation association was only partially supported. Settlement type significantly moderated the deprivation-depression association: the quadratic association was strongest in cities and attenuated, or shifted towards a concave pattern, in less densely populated settlement types. This settlement-related moderation was additionally associated with income, particularly in urban and rural counties, and less consistently in sparsely populated areas.

**Conclusions:**

The psychological burden of socioeconomic deprivation is not uniform, but differs systematically by settlement context and, less consistently, by individual income. These associations were most pronounced, and most clearly evident, in major cities. Addressing mental health inequalities linked to neighbourhood deprivation may therefore benefit from strategies tailored to settlement-specific conditions, alongside broader efforts aimed at individual socioeconomic resources.

**Supplementary Information:**

The online version contains supplementary material available at https://doi.org/10.1186/s12889-026-30253-8.

## Introduction

Mental health is increasingly recognised as being influenced not only by individual characteristics but also by the conditions of the environments in which people live. Research in spatial epidemiology and public health has long established that place plays a crucial role in shaping health outcomes, including mental wellbeing [1]. Environmental influences are not merely background factors but shape everyday experience: neighbourhood conditions, ranging from material deprivation and institutional access to safety and green space, are directly and indirectly associated with psychological vulnerability [2]. We focus specifically on depressive symptoms, given that depression remains one of the leading contributors to disability worldwide and is particularly sensitive to social and environmental conditions [3].

Existing research on spatial and environmental determinants of mental health has often examined contextual characteristics and individual socioeconomic resources as separate or independent predictors, with less consistent attention to their joint, nonlinear, or interactive relationships [4, 5]. This limitation is important because the mental-health consequences of living in a socioeconomically disadvantaged area may depend on the resources available to individuals and on the broader settlement context. While recent multilevel evidence from Germany demonstrates independent effects of area-level deprivation and individual socioeconomic status on depressive symptoms [6], analyses that consider area-level deprivation, individual socioeconomic status, and settlement type simultaneously remain limited.

This gap is particularly evident in studies of spatial inequality, where neighbourhood context and personal resources are frequently analysed separately [4]. However, environmental disadvantage is not experienced in isolation. Its psychological impact depends on how structural constraints interact with personal capacity, social position, and the physical form of the place itself [7, 8].

This study addresses this gap by examining how depressive symptoms are shaped by the interplay between neighbourhood conditions, household income, and settlement structure. Drawing on data from the German Health Update 2019/2020 wave of the European Health Interview Survey (GEDA 2019/2020-EHIS) [9], we analyse how these factors combine to shape the psychological consequences of living in more or less advantaged environments. Instead of treating neighbourhood deprivation, income, and urban form as separate predictors, we consider them as interdependent dimensions of people’s lived environment, each shaping the distribution of mental health vulnerability.

### Conceptual background

This study draws on the person-in-environment perspective proposed by Wapner and Demick [10], which holds that individuals and environments are inseparably linked. Mental health is understood here not as a purely individual state, but as the outcome of continuous interaction between people and their surroundings. On the personal side, Wapner and Demick distinguish three interrelated levels: the physical (e.g., physical health), the psychological-intrapersonal (e.g., emotional states), and the sociocultural (e.g., social roles). The environment is similarly structured into physical (e.g., built and natural environment), psychological-interpersonal (e.g., relationships), and sociocultural (e.g., values, norms, deprivation) domains [10].

**Fig. 1:**
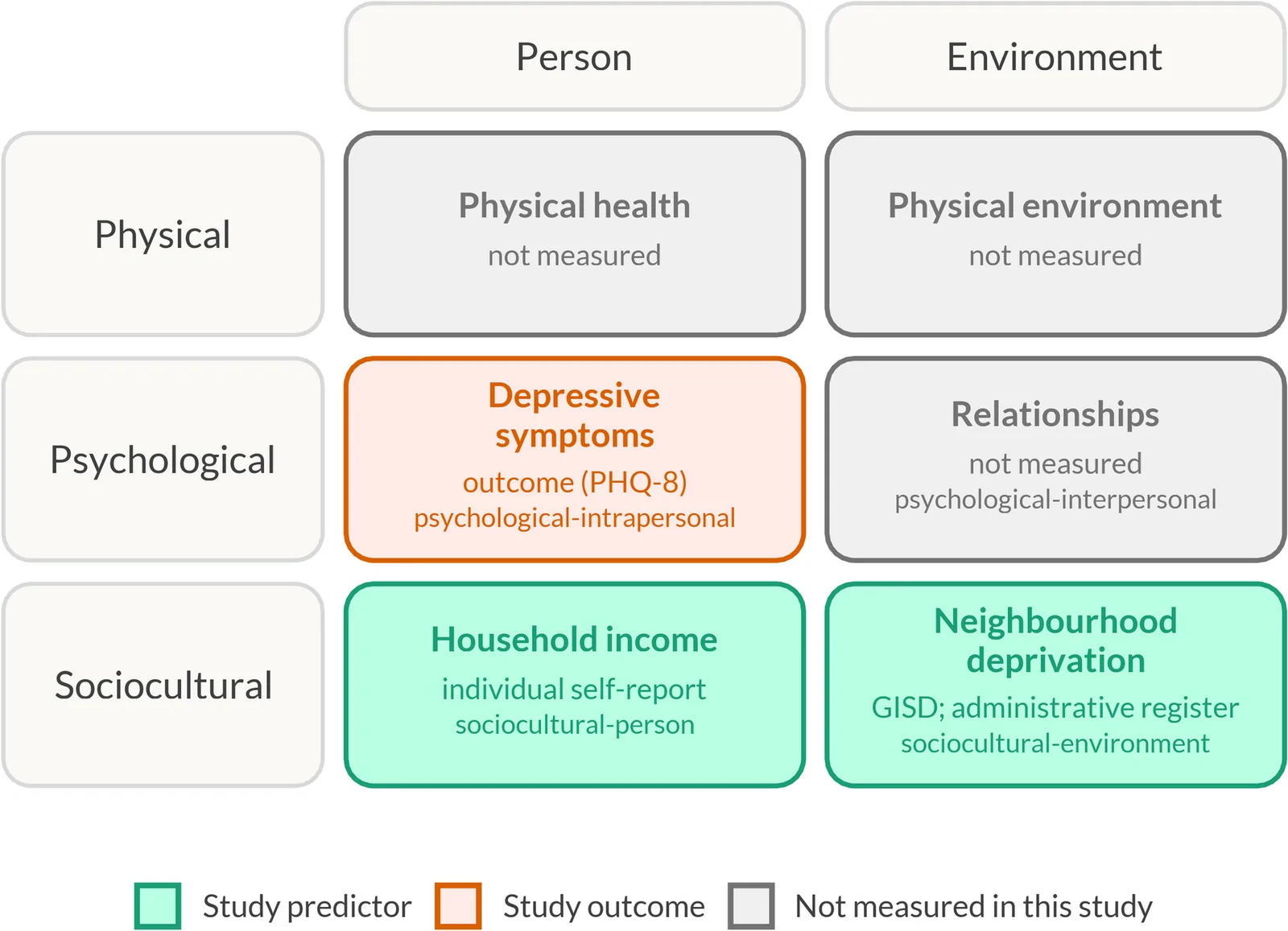
Mapping of study variables onto the person-in-environment framework. Note. Study variables are mapped onto the person-in-environment framework [10]. Green cells indicate variables included as predictors in this study (household income, neighbourhood deprivation); the orange cell indicates the study outcome (depressive symptoms); grey cells indicate framework dimensions not measured in this study. Settlement type is not mapped onto a single environmental level; it structures the geographic scale at which these levels are stratified in subsequent analyses (see Methods). GISD = German Index of Socioeconomic Deprivation; PHQ-8 = Patient Health Questionnaire-8

Within this framework, mental health emerges from the mutual influence of personal and environmental dimensions. Experiences of stress, control, safety, or belonging arise from the way individuals relate to the material, social, and symbolic structures around them [10]. For the purposes of this study, neighbourhood deprivation (as measured by the German Index of Socioeconomic Deprivation [11]) reflects the sociocultural environment, given that the GISD is composed of area-level education, employment, and income indicators rather than physical or infrastructural characteristics. Household income indicates the person’s social position within that same sociocultural environment. Depressive symptoms are treated as an outcome within the psychological-intrapersonal domain (see Measures). Figure 1 summarises this mapping.

This model contrasts with unifactorial explanations such as Evans’ [12] environmental stress theory, which emphasises the physiological burden of environmental exposure but does not capture the reciprocal dynamics between people and place. Similarly, while Caplan’s [13] person–environment fit theory draws attention to individual coping strategies, it assumes relatively stable contexts and gives limited attention to structural constraints. In contrast, the person-in-environment [10] approach accounts for ongoing interaction, spatial variation, and social embeddedness—factors that are crucial for understanding the lived experience of deprivation, while opening the possibility of nonlinear influences of the environment. In this study, such contextual variation is captured through regional deprivation and settlement type rather than through spatially explicit modelling or measures of social embeddedness (see Limitations).

The empirical relevance of this perspective is underscored by several studies. Stafford and Marmot [14] demonstrated that neighbourhood deprivation predicts psychological distress even after adjusting for personal socioeconomic status, highlighting the independent role of structural conditions. Miles et al. [15] showed that the quality of the built and social environment shapes mental health by influencing how individuals interpret and cope with stress. Tomita et al. [16], in a study from South Africa, found that residential greenness reduces the incidence of depression even after controlling for poverty, supporting the protective role of the local physical environment; see also Kley and Dovbishchuk [17].

### Spatial mental health inequalities in Europe and Germany

Across Europe, depression is not only socially but also spatially stratified. A global meta-analysis shows that the odds of depression are about 30% higher in urban areas of high-income countries than in rural ones [5]. A nine-country study found that mental health disparities are greatest among socioeconomically disadvantaged residents, especially when poor housing conditions are present [18]. Longitudinal data from the UK further show that access to urban green space reduces psychological distress—particularly in highly deprived neighbourhoods [19–21].

In Germany, these spatial disparities persist despite universal healthcare. A multicity study in the Ruhr region found that high neighbourhood deprivation, urban density, and low perceived safety were associated with higher PHQ-9 (Patient Health Questionnaire-9) scores [22]. Nevertheless, research has only inconsistently integrated individual socioeconomic characteristics with multiple dimensions of neighbourhood context and the built environment, and cross-level interactions between these factors remain comparatively understudied [23].

### Aim of the study

While the associations between neighbourhood deprivation, household income, and depressive symptoms are well documented individually, less is known about whether and how these associations vary systematically across settlement structures. This study investigates how depressive symptoms in Germany relate to the interplay between neighbourhood deprivation, household income, and settlement structure. It builds on the idea that mental health is not merely the sum of individual and environmental factors, but emerges from how people live within, respond to, and are constrained by their surroundings. Theoretical models suggest that neighbourhood effects are unlikely to be linear; instead, critical thresholds may exist where the accumulation of social and physical disorder disproportionately impacts mental health, often manifesting as accelerating (quadratic) effects in highly deprived areas [24, 25]. Consequently, we are particularly interested in whether the psychological burden of spatial disadvantage follows a linear gradient, or whether it becomes disproportionately severe in highly deprived areas; whether income serves as a buffer; and whether these dynamics differ between urban versus rural environments. This study addresses this gap by testing not only the overall pattern of association (H1–H3), but also whether settlement structure moderates both the deprivation–depression association (H4) and its buffering by household income (H5), using formal interaction tests rather than descriptive comparison alone.

Based on this theoretical and empirical foundation, our hypotheses are:

- H1: There is a non-linear (quadratic) association between neighbourhood socioeconomic deprivation and depressive symptoms, with disproportionately higher symptom severity in highly deprived areas.
- H2: Household equivalent income is inversely associated with depressive symptoms, replicating established income-related disparities in mental health outcomes.
- H3: Household equivalent income moderates the relationship between deprivation and depressive symptoms; the negative effects of deprivation are strongest at lower income levels.
- H4: The association between neighbourhood deprivation and depressive symptoms is stronger in urban areas compared to rural or sparsely populated areas.
- H5: The income-based buffering of neighbourhood deprivation is stronger in urban environments, where socioeconomic disparities and stressors are more pronounced.

## Methods

### Sample

To investigate our hypotheses, we employed data from the GEDA 2019/2020-EHIS [9] survey conducted by the Robert Koch Institute (RKI). GEDA is a cross-sectional survey managed by the RKI on behalf of the German Federal Ministry of Health, with surveys being carried out at multi-year intervals. As a fixed component of the RKI’s health monitoring system for Germany since 2008, GEDA contributes significantly to understanding public health. The GEDA 2019/2020-EHIS was administered through computer-assisted telephone interviewing (CATI), using dual-frame random sampling of landline and mobile numbers, with a response rate of 21.6% and representativeness established via post-stratification weighting [9].

The GEDA19 survey involved 23,001 participants. For our analyses, we excluded individuals with missing values on the PHQ-8 scale, those without data on neighbourhood socioeconomic deprivation, and those under the age of 18. Following this stepwise exclusion, our analysis sample consisted of 20,773 individuals.

The average age of the sample was 56.36 years (*SD* = 17.28 years). Approximately 52.5% of the sample were female (*n* = 10,904). Regarding employment status, 53.9% (*n* = 11,207) of individuals reported being employed. About 2.1% (*n* = 438) indicated they were unable to work due to long-term health problems. The average net equivalent household income was 2,296.70 Euro per month (*SD* = 1,396.82 Euro). Missing values on income were imputed using single stochastic regression imputation, in which predicted values from a regression model were combined with a random error term to preserve natural variability, following RKI-standard procedures [26], and income was subsequently categorised into quintiles. Approximately 11.9% (*n* = 2,472) of individuals belonged to the lowest (1st ) household equivalent income quintile, while 29.8% (*n* = 6,194) belonged to the highest (5th ) household equivalent income quintile (HeI). A detailed description of the sample characteristics can be found in Table 1.

**Table 1:** Sociodemographic and neighbourhood description of the sample

Variable	
Age in years [*M* (*SD*)]	56.36 (17.28)
Sex [*n* (%)]	Male: 9,869 (47.5%) Female: 10,904 (52.5%)
Migration Status [*n* (%)]	Migrants: 1,989 (9.6%) non-Migrants: 18,694 (90.0%) NA: 90 (0.4%)
Net household equivalent income in Euro [*M* (*SD*)]	2,296.70 (1396.82)
Household equivalent income in quintiles [*n* (%)]	1st quintile: 2,472 (11.9%) 2nd quintile: 3,704 (17.8%) 3rd quintile: 3,547 (17.1%) 4th quintile: 4,856 (23.4%) 5th quintile: 6,194 (29.8%)
Living with Partner [*n* (%)]	Living with a Partner: 12,470 (60.0%) Living without a Partner: 8,240 (39.7%) NA: 63 (0.3%)
Household type [*n* (%)]	Single Person Household: 6,179 (29.7%) Single Parent with Children Household: 726 (3.5%) Couple with no Children Household: 7,686 (37.0%) Couple with Children Household: 4,196 (20.2%) Other types of Households: 1,710 (8.2%) NA: 276 (1.3%)
German Socioeconomic Deprivation Index [*n* (%)]	Low Deprivation: 3,526 (17%) 2: 1,401 (6.7%) 3: 1,886 (9.1%) 4: 2,023 (9.7%) 5: 1,293 (6.2%) 6: 3,796 (18.3%) 7: 2,123 (10.2%) 8: 1,288 (6.2%) 9: 2,000 (9.6%) Highest Deprivation: 1,437 (6.9%)
Employment Status [*n* (%)]	Unemployed: 336 (1.6%) Unemployed for other Reasons: 212 (1.0%) Retired: 7,305 (35.2%) Unable to work (long-term health problems): 438 (2.1%) Employed: 11,207 (53.9%) Student: 750 (3.6%) Homemaker: 480 (2.3%) Military or Civil Service: 16 (0.1%) NA: 29 (0.1%)
County Type [*n* (%)]	Cities: 7,406 (36%) Urban County: 8,135 (39%) Rural County: 2,741 (13%) Sparsely Populated Rural County: 2,491 (12%)

Values represent descriptive characteristics of the analytic sample (N = 20,773). Percentages may not add up to 100% due to rounding. NA indicates missing values. Sex was coded as 1 = male and 2 = female. Migration status was coded as 1 = migrants and 2 = non-migrants

*GISD* German Index of Socioeconomic Deprivation

### Assessing mental health and neighbourhood characteristics

In this section, we describe the instruments used to measure our study’s key variables: depressive symptoms (outcome); socioeconomic deprivation and county structural characteristics (exposures); and individual-level covariates (including age, sex, unemployment, and others) to adjust for compositional effects.

#### Depressive symptoms

Depressive symptoms were assessed using the eight-item version of the Patient Health Questionnaire (PHQ-8) [27].The ninth item assessing suicidal and self-harming behaviour was excluded, consistent with standard practice for the PHQ-8 in population-based telephone surveys, where adequate clinical follow-up cannot be guaranteed [27]. The Participants reported the frequency of depressive symptoms in the last two weeks on a four-point scale. Response options ranged from “not at all” (0), “on several days” (1), “on more than half the days” (2), or “nearly every day” (3). Individuals could thus score between 0 and 24, with values at and above the cut-off value of ≥ 10 indicating depressive symptoms [27].

#### Socioeconomic deprivation of the neighbourhood

Socioeconomic deprivation was assessed using the German Index of Socioeconomic Deprivation (GISD). The GISD is a measure of regional socioeconomic deprivation developed by the RKI to investigate associations between social disadvantage and health [11]. The GISD consists of the subdimensions of education, employment, and income, and a principal component analysis was used to weight the individual subscales for the index. A detailed description and validation can be found in the work of Michalski and colleagues [11].

A high GISD score indicates high socioeconomic deprivation, while a low GISD score indicates low socioeconomic deprivation in the region. In this study, we used GISD data for all 401 counties in Germany, which were matched to respondents based on their place of residence. To ensure compliance with data protection regulations, we rely on the deprivation deciles, with higher deciles corresponding to greater levels of regional socioeconomic deprivation. A detailed distribution of GISD deciles within the sample is presented in Table 1.

#### Settlement structures of german counties

Settlement type was classified using the official typology from the BBSR [Bundesinstitut für Bau-, Stadt- und Raumforschung]. The 401 German counties were assigned to one of four settlement structures. One group consists of major cities (hereafter referred to as cities) with a minimum of 100,000 inhabitants, while the remaining 361 counties are classified into urban counties (Urban), rural counties (Rural), and sparsely populated rural counties (Sparsely Populated) based on the following criteria [28]: (1) proportion of the population in large and medium-sized cities, (2) population density in the county, (3) population density in the county excluding large and medium-sized cities (exact thresholds provided in Supplement A). A visual overview of these settlement structures is presented in the supplementary materials (Figure A1). The distribution of study participants across the four categories is shown in Table 1.

### Statistical analysis

The data were analysed using R-Studio [29], and a detailed list of the packages used can be found in the supplement (Supplement B1).

First, the descriptive evaluations were carried out; in addition to the frequencies, mean values and standard deviations were calculated. The correlations were then calculated using Pearson’s r. Values less than 0.20 indicate a weak correlation, values between 0.20 and 0.30 indicate a moderate correlation, and values greater than 0.30 indicate a strong correlation [30, 31].

Before addressing the main research question, we assessed the functional form of key predictors using LOESS smoothing [32] to guide model specification without altering a priori hypotheses. Comparing non-parametric LOESS curves to linear OLS fits revealed a clear divergence for neighbourhood deprivation, whose association with depressive symptoms departed from linearity, particularly at higher levels of deprivation, whereas household income showed a largely linear association across its range (Supplement Figure B2). This divergence provided the empirical basis for adopting a quadratic specification for deprivation in all subsequent models; a cubic term was also tested but did not improve model fit and was therefore not retained, in the interest of parsimony (Supplement B2). COVID-period effects (2019 vs. 2020) were checked, but associations remained stable (Supplement B3).

All regression analyses were estimated using population weights provided by the RKI, based on post-stratification by age, sex, region, and education to reflect the composition of the German population [9]. Based on the specification test, we conducted polynomial regression analyses, with GISD mean-centred prior to computing linear and quadratic terms. This is standard practice in polynomial regression, as it reduces non-essential collinearity between lower- and higher-order terms and renders the linear coefficient interpretable at the observed sample mean rather than at the unobserved value of zero [33, 34]. We report unstandardized regression coefficients (*B*, in PHQ-8 raw points) along with 95% confidence intervals and significance levels, computed directly from the fitted weighted models. We opted against standardized coefficients, as post-hoc standardization via model refitting did not reliably propagate survey weights in models containing polynomial and interaction terms; unstandardized coefficients also offer more direct clinical interpretability given the PHQ-8’s established raw-score metric. Variance inflation factors confirmed no problematic collinearity between GISD and household income (Supplement B4). Analyses were first conducted for the full sample across Germany (Model 1). To formally test whether settlement structure moderates these associations, we compared Model 1 to nested models additionally including settlement type and its interaction with deprivation (H4), and settlement type’s interaction with both deprivation and income jointly (H5), using weighted F-tests.

To examine whether the associations between key predictors and depressive symptoms vary across settlement types, Model 1 additionally included an interaction term between GISD and net equivalent household income, to assess the joint association of area-level deprivation and individual-level resources, alongside sociodemographic controls, including sex, age, household type, migration status, and employment status, to account for individual-level variation.

## Results

Before testing our hypotheses, we first examined descriptive statistics for the key variables. Depressive symptoms, measured using the PHQ-8, showed an overall mean score of 2.99 (SD = 3.46) across Germany. Average scores varied only modestly across settlement types, from 2.87 (SD = 3.42) in urban counties to 3.12 (SD = 3.46) in cities, with rural counties (M = 3.01, SD = 3.62) and sparsely populated counties (M = 2.99, SD = 3.42) falling in between (Table 2). These raw group means reflect unadjusted symptom levels and should not be interpreted as indicating where deprivation is most strongly associated with depressive symptoms; this question is addressed directly in the regression analyses below.

**Table 2:** Mean and SD of PHQ-8 by county structures and total

	*N*	PHQ-8 M (SD)	Skew	Kurtosis	SE
Cities	7,406	3.12 (3.46)	1.90	4.74	0.04
Urban Counties	8,135	2.87 (3.42)	2.15	6.13	0.04
Rural Counties	2,741	3.01 (3.62)	2.13	5.63	0.07
Sparsely Populated Rural Counties	2,491	2.99 (3.42)	1.85	4.28	0.07
Total	20,773	2.99 (3.46)	2.02	5.34	0.02

*PHQ-8* Patient Health Questionnaire-8, *SE* Standard Error; *M*  Mean, *SD* Standard Deviation

Table 3 presents the correlations among the key variables. Additional analyses stratified by county structure are reported in the supplementary materials (Supplement C). Household equivalent income was significantly negatively correlated with PHQ-8 scores (*r* = –.18, *p* < .001), whereas GISD showed only a weak positive correlation with PHQ-8 (*r* = .02, *p* < .01) and a weak negative correlation with income (*r* = –.11, *p* < .001), indicating limited conceptual overlap between the two predictors.

**Table 3:** Correlation matrix of the key variables

	1. PHQ-8	2. Sex	3. Age	4.Migration Status	5. HeI
1. PHQ-8					
2. Sex	0.107^***^				
3. Age	− 0.049^***^	0.062^***^			
4. Migration Status	− 0.049^***^	0.010	0.144^***^		
5. HeI	− 0.179^***^	− 0.104^***^	0.025^***^	0.083^***^	
6. GISD	0.023^**^	− 0.002	0.088^***^	0.080^***^	− 0.107^***^

Correlations were computed using Pearson’s r with pairwise deletion. Age was coded such that higher values indicate older age

*PHQ-8* Patient Health Questionnaire-8, *GISD* German Index of Socioeconomic Deprivation, *HeI* Household equivalent Income quintiles

*p* < .05 = *, *p* < .01 = **, *p* < .001 = ***

The regression model for the full sample explained 12.8% of the variance in depressive symptoms (adjusted *R²* = 0.127) and was statistically significant, *F*(29, 20,318) = 103, *p* < .001. Before turning to the hypothesis tests, we briefly note the direction of the sociodemographic covariates. As shown in Table 4, sociodemographic and socioeconomic covariates showed associations consistent with prior research: women, individuals living alone, those without employment, those unable to work due to long-term health problems, and those with a migration background reported higher depressive symptoms (all *p* < .001; full estimates in Table 4). As these associations are well established and included primarily as covariates, we do not discuss them further; our focus is on whether neighbourhood deprivation and its interplay with income remain associated with depressive symptoms once these individual-level factors are accounted for.

Consistent with H1, neighbourhood socioeconomic deprivation demonstrated a significant non-linear association with depressive symptoms. The linear GISD term was not significant (B = –0.01, *p* = .690), but the quadratic term was (B = 0.02, *p* = .011), indicating an upward-curving relationship in which depressive symptoms increase disproportionately in highly deprived areas (see Table 4).

H2 was supported by a pronounced protective association between household income and depressive symptoms. Relative to the lowest income quintile, symptom levels declined progressively across the second (B = –0.42, *p* < .001), third (*B* = –0.60, *p* < .001), fourth (B = − 1.07, *p* < .001), and fifth quintiles (B = − 1.38, *p* < .001), reflecting a clear socioeconomic gradient in mental health (see Table 4).

Finally, H3 proposed that income is associated with weaker associations between neighbourhood deprivation and depressive symptoms. The results offer only partial and mixed support for this hypothesis. Although linear interaction terms were non-significant, a significant quadratic interaction emerged for the third income quintile relative to the first (B = –0.03, *p* = .014), indicating that the two groups’ associations between GISD and depressive symptoms differ in shape. However, this pattern was not part of a monotonic income gradient: the second, fourth, and fifth quintiles, including the highest-income group, where a buffering effect would be expected to be most pronounced, did not differ significantly from the reference group (Table 4). We interpret this cautiously in the Discussion below.

**Table 4:** Weighted regression model predicting depressive symptoms (PHQ-8) – Model 1

Predictors	PHQ-8		
	Estimates	CI	*p*
Intercept	7.04	6.48–7.61	< 0.001
Female	0.63	0.53 – 0.74	< 0.001
Age	− 0.02	− 0.02 – − 0.01	< 0.001
Household Type [Single parent with child]	− 0.48	− 0.77 – − 0.18	0.001
Household Type [Couple no children]	− 0.55	− 0.95 – − 0.15	0.007
Household Type [Couple with children]	− 0.96	-1.37 – − 0.55	< 0.001
Household Type [Other type of household]	− 0.70	− 0.91 – − 0.48	< 0.001
Living without partner	0.08	− 0.30 – 0.47	0.671
Employment [Not employed for other reasons]	0.12	− 0.41 – 0.65	0.667
Employment [Retired]	-1.80	-2.14 – -1.47	< 0.001
Employment [Unable to work due to long-term health problems]	3.51	3.10–3.91	< 0.001
Employment [Employed]	-1.81	-2.11 – -1.50	< 0.001
Employment [Student]	-1.70	-2.07 – -1.32	< 0.001
Employment [Homemaker]	-1.75	-2.19 – -1.32	< 0.001
Employment [Military or Civil Service]	− 0.82	-2.47 – 0.82	0.326
Without Migration Background	− 0.62	− 0.78 – − 0.47	< 0.001
Household Equivalent Income [2nd quint.]	− 0.42	− 0.65 – − 0.18	< 0.001
Household Equivalent Income [3rd quint.]	− 0.60	− 0.85 – − 0.35	< 0.001
Household Equivalent Income [4th quint.]	-1.07	-1.31 – − 0.82	< 0.001
Household Equivalent Income [5th quint.]	-1.38	-1.63 – -1.13	< 0.001
Deprivation	− 0.01	− 0.05 – 0.03	0.690
Deprivation [quadratic]	0.02	0.00 – 0.04	0.011
Household Equivalent Income [2] × Deprivation	0.03	− 0.02 – 0.09	0.263
Household Equivalent Income [3] × Deprivation	0.04	− 0.02 – 0.10	0.148
Household Equivalent Income [4] × Deprivation	0.00	− 0.06 – 0.06	0.944
Household Equivalent Income [5] × Deprivation	− 0.01	− 0.07 – 0.05	0.734
Household Equivalent Income [2] × Deprivation [quadratic]	− 0.02	− 0.04 – 0.01	0.146
Household Equivalent Income [3] × Deprivation [quadratic]	− 0.03	− 0.05 – − 0.01	0.014
Household Equivalent Income [4] × Deprivation [quadratic]	− 0.01	− 0.04 – 0.01	0.179
Household Equivalent Income [5] × Deprivation [quadratic]	− 0.01	− 0.03 – 0.01	0.392
Observations	20,348		
*R*^*2*^ / *R*^*2*^ adjusted	0.128 / 0.127		

Reference Group for Female is Male; Reference Group Household Type is Single Person Household; Reference for Living without Partner is Living with Partner; Reference for Employment status is Unemployed; Reference for Household Equivalent Income is under 1st quintile; Reference for Without Migration Background is With Migration Background

**Fig. 2:**
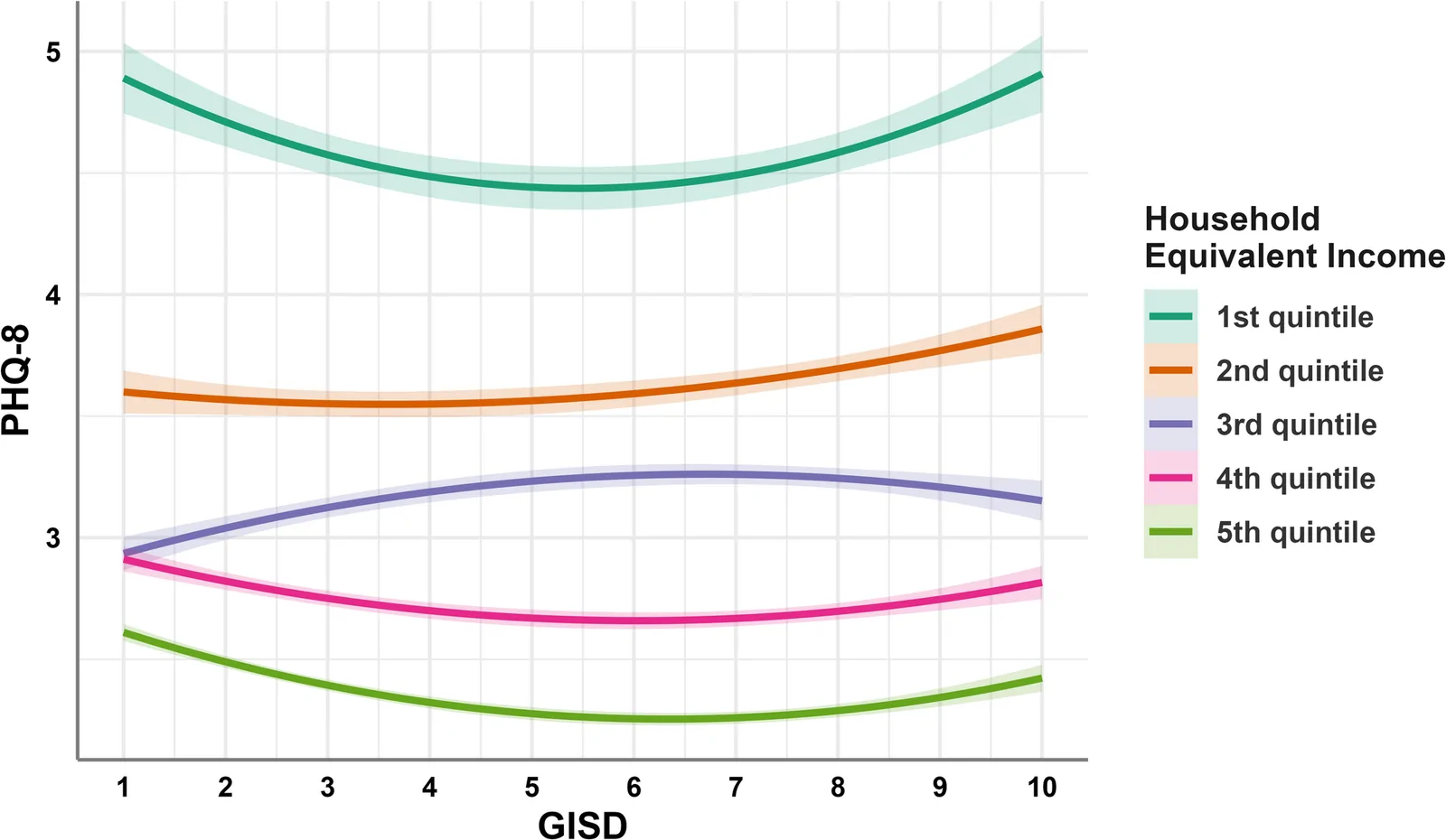
Polynomial interaction effects for the full German sample. Note. Predicted values of depressive symptoms (PHQ-8 = Patient Health Questionnaire 8) across levels of neighbourhood socioeconomic deprivation (GISD = German Index of Socioeconomic Deprivation), stratified by household equivalent income quintiles. Lines represent polynomial fits with 95% confidence intervals

To further illustrate these associations, Fig. 2 shows the model-predicted relationship between neighbourhood deprivation and depressive symptoms across income quintiles for the full German sample. Across most quintiles, predicted PHQ-8 scores followed a broadly similar U-shaped pattern, declining at low-to-moderate levels of deprivation before rising again at higher levels, suggesting that the association between neighbourhood deprivation and depressive symptoms is descriptively present across income groups, even though income levels differ markedly. The third income quintile stood out as an exception, following a distinctly different, non-U-shaped trajectory compared to the other four quintiles.

To formally test whether the association between neighbourhood deprivation and depressive symptoms varies by settlement type (H4), we compared Model 1 to a nested model additionally including settlement type and its interaction with neighbourhood deprivation (linear and quadratic terms). This extended model fit the data significantly better than Model 1, *F*(9, 20309) = 5.97, *p* < .001, indicating that the association between neighbourhood deprivation and depressive symptoms differs across settlement types.

Consistent with H4, the quadratic association between neighbourhood deprivation and depressive symptoms was significant in cities, the reference category (B = 0.05, *p* < .001). This association was significantly weaker in urban counties (B = –0.03, *p* < .001), rural counties (B = –0.06, *p* < .001), and sparsely populated areas (B = –0.06, *p* < .001; Table 5; full table in Supplement Table D1). Rather than a uniform attenuation, this reflects a shift in the shape of the association. The curvature remained upward-opening only in cities. In rural and sparsely populated areas, it reversed toward a concave pattern, and in urban counties it flattened.

**Table 5:** Settlement-related coefficients from the weighted regression model testing H4

Predictors	PHQ-8		
	Estimates	CI	*p*
Deprivation	0.03	− 0.02 – 0.07	0.257
Deprivation [quadratic]	0.05	0.03 – 0.06	< 0.001
Settlement Type [Urban County]	0.20	0.01 – 0.39	0.042
Settlement Type [Rural County]	0.41	0.18 – 0.64	0.001
Settlement Type [Sparsely Populated Rural County]	0.21	− 0.04 – 0.47	0.104
Settlement Type [Urban County] × Deprivation	− 0.04	− 0.08 – 0.01	0.132
Settlement Type [Rural County] × Deprivation	− 0.07	− 0.13 – − 0.01	0.022
Settlement Type [Sparsely Populated Rural County] × Deprivation	0.06	− 0.02 – 0.13	0.120
Settlement Type [Urban County] × Deprivation [quadratic]	− 0.03	− 0.05 – − 0.02	< 0.001
Settlement Type [Rural County] × Deprivation [quadratic]	− 0.06	− 0.08 – − 0.03	< 0.001
Settlement Type [Sparsely Populated Rural County] × Deprivation [quadratic]	− 0.06	− 0.08 – − 0.03	< 0.001
Observations	20,348		
R² / R² adjusted	0.131 / 0.129		

Reference category for Settlement Type is Cities. Model compared to Model 1 via nested F-test, *F*(9, 20309) = 5.97, *p* < .001. Sociodemographic and income covariates omitted from this table for brevity; full model reported in Supplement Table D1

**Fig. 3:**
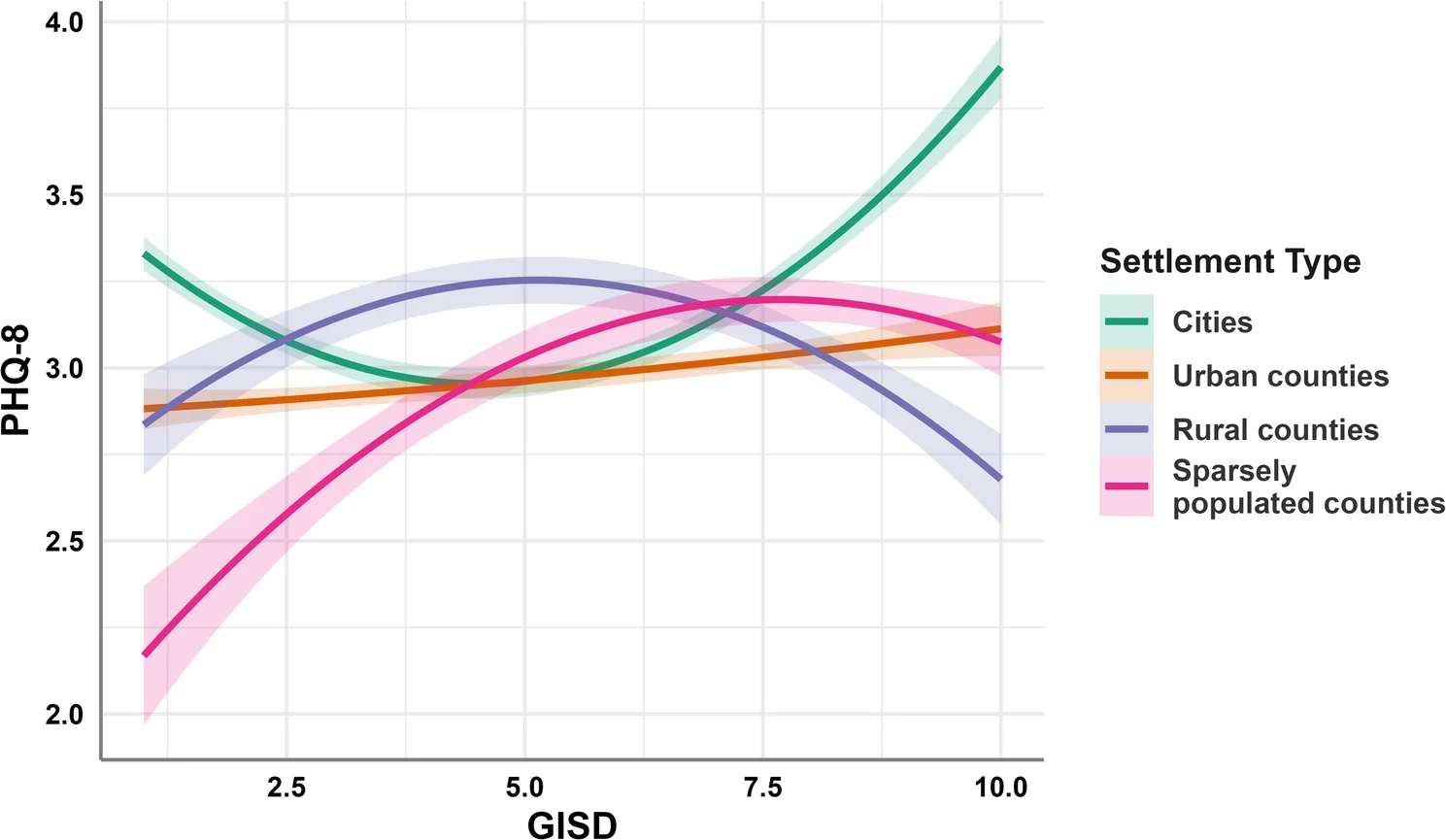
Polynomial interaction effects by settlement type. Note. Predicted values of depressive symptoms (PHQ-8 = Patient Health Questionnaire 8) across levels of neighbourhood socioeconomic deprivation (GISD = German Index of Socioeconomic Deprivation), stratified by settlement type. Lines represent polynomial fits with 95% confidence intervals

Figure 3 illustrates this pattern graphically. In cities, predicted PHQ-8 scores followed a clear U-shaped trajectory, declining at low levels of deprivation before rising sharply toward the highest deprivation decile. Urban counties showed a relatively flat, only slightly increasing trajectory across the GISD range. Rural counties and sparsely populated areas, by contrast, followed an opposite pattern, with predicted symptoms rising toward moderate levels of deprivation before levelling off or declining again at the highest deprivation decile.

To formally test whether this settlement-related moderation is further associated with income (H5), we compared the H4 model to a nested model additionally including the three-way interaction between settlement type, income, and neighbourhood deprivation (linear and quadratic terms). This extended model fit the data significantly better than the H4 model, *F*(36, 20273) = 2.93, *p* < .001, indicating that the extent to which settlement type moderates the deprivation–depression association additionally varies by income.

Table 6 (full table in Supplement Table D2) presents the full set of three-way interaction terms testing whether the settlement-related moderation identified above (H4) is additionally associated with income. In urban and rural counties, all four income-by-settlement quadratic interaction terms differed significantly from the reference group (cities, first income quintile; all *p* < .01), indicating a robust income-dependent pattern in both settlement types. In sparsely populated areas, only two of the four quadratic terms reached significance, providing comparatively weaker and less consistent evidence of income-dependent moderation in this settlement type.

**Table 6:** GISD main effects and three-way interaction coefficients from the weighted regression model testing H5

Predictors	PHQ-8		
	Estimates	CI	*p*
Deprivation, Cities & 1st quintile [reference]	0.11	0.04 – 0.17	0.001
Deprivation [quadratic], Cities & 1st quintile [reference]	0.10	0.07 – 0.12	< 0.001
Household Equivalent Income [2] × Settlement Type [Urban County] × Deprivation	0.41	0.27 – 0.56	< 0.001
Household Equivalent Income [3] × Settlement Type [Urban County] × Deprivation	0.24	0.09 – 0.40	0.002
Household Equivalent Income [4] × Settlement Type [Urban County] × Deprivation	0.27	0.12 – 0.42	< 0.001
Household Equivalent Income [5] × Settlement Type [Urban County] × Deprivation	0.26	0.11 – 0.41	0.001
Household Equivalent Income [2] × Settlement Type [Rural County] × Deprivation	− 0.10	− 0.30 – 0.09	0.290
Household Equivalent Income [3] × Settlement Type [Rural County] × Deprivation	− 0.17	− 0.37 – 0.03	0.097
Household Equivalent Income [4] × Settlement Type [Rural County] × Deprivation	− 0.09	− 0.28 – 0.10	0.353
Household Equivalent Income [5] × Settlement Type [Rural County] × Deprivation	− 0.11	− 0.30 – 0.08	0.267
Household Equivalent Income [2] × Settlement Type [Sparsely Populated Rural County] × Deprivation	0.24	− 0.00 – 0.49	0.054
Household Equivalent Income [3] × Settlement Type [Sparsely Populated Rural County] × Deprivation	0.07	− 0.19 – 0.32	0.602
Household Equivalent Income [4] × Settlement Type [Sparsely Populated Rural County] × Deprivation	− 0.06	− 0.30 – 0.18	0.617
Household Equivalent Income [5] × Settlement Type [Sparsely Populated Rural County] × Deprivation	0.03	− 0.22 – 0.28	0.793
Household Equivalent Income [2] × Settlement Type [Urban County] × Deprivation [quadratic]	0.12	0.06 – 0.17	< 0.001
Household Equivalent Income [3] × Settlement Type [Urban County] × Deprivation [quadratic]	0.10	0.04 – 0.16	0.001
Household Equivalent Income [4] × Settlement Type [Urban County] × Deprivation [quadratic]	0.09	0.03 – 0.15	0.002
Household Equivalent Income [5] × Settlement Type [Urban County] × Deprivation [quadratic]	0.12	0.06 – 0.17	< 0.001
Household Equivalent Income [2] × Settlement Type [Rural County] × Deprivation [quadratic]	0.15	0.08 – 0.22	< 0.001
Household Equivalent Income [3] × Settlement Type [Rural County] × Deprivation [quadratic]	0.16	0.08 – 0.23	< 0.001
Household Equivalent Income [4] × Settlement Type [Rural County] × Deprivation [quadratic]	0.15	0.08 – 0.22	< 0.001
Household Equivalent Income [5] × Settlement Type [Rural County] × Deprivation [quadratic]	0.17	0.10 – 0.24	< 0.001
Household Equivalent Income [2] × Settlement Type [Sparsely Populated Rural County] × Deprivation [quadratic]	0.08	0.00 – 0.16	0.047
Household Equivalent Income [3] × Settlement Type [Sparsely Populated Rural County] × Deprivation [quadratic]	0.06	− 0.02 – 0.15	0.150
Household Equivalent Income [4] × Settlement Type [Sparsely Populated Rural County] × Deprivation [quadratic]	0.11	0.03 – 0.19	0.009
Household Equivalent Income [5] × Settlement Type [Sparsely Populated Rural County] × Deprivation [quadratic]	0.08	− 0.00 – 0.17	0.056
Observations	20,348		
R² / R² adjusted	0.135 / 0.132		

Deprivation and Deprivation [quadratic] main effects apply to the joint reference group (Cities, 1st income quintile). Remaining rows show the full three-way interaction between income quintile, settlement type, and neighbourhood deprivation. Model compared to H4 model via nested F-test, *F*(36, 20273) = 2.93, *p* < .001. Sociodemographic covariates, two-way interaction terms, and main effects omitted from this table for brevity; full model reported in Supplement Table D2

**Fig. 4:**
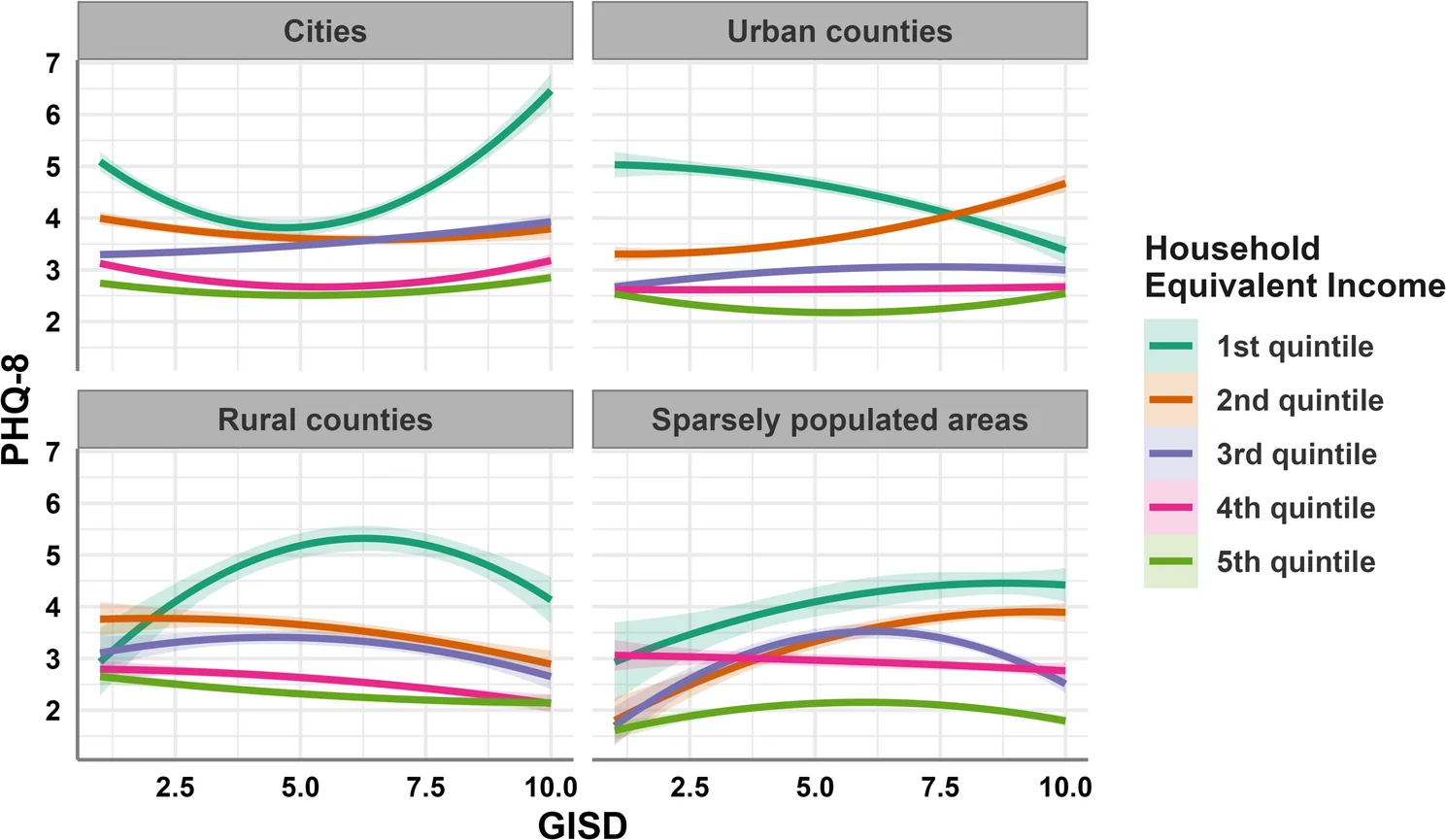
Polynomial interaction effects by settlement type and income quintile. Note. Predicted values of depressive symptoms (PHQ-8 = Patient Health Questionnaire 8) across levels of neighbourhood socioeconomic deprivation (GISD = German Index of Socioeconomic Deprivation), stratified by settlement type and household equivalent income quintile. Lines represent polynomial fits with 95% confidence intervals

In cities, the three-way interaction confirmed that only the lowest income quintile retained the pronounced U-shaped association with deprivation identified in H1 and H4 (Fig. 4). Predicted symptoms declined at low levels of deprivation before rising sharply toward the highest deprivation decile, whereas the second through fifth income quintiles showed relatively flat patterns across the same range.

In urban counties, the pattern differed from cities. The second income quintile showed a marked increase in predicted symptoms with rising deprivation, exceeding the first quintile at high deprivation levels, while the first quintile itself declined gradually rather than following a U-shape. The third, fourth, and fifth quintiles remained comparatively stable across the deprivation range.

In rural counties, the direction of the interaction reversed relative to cities. The lowest income quintile showed an inverted U-shaped pattern, with predicted symptoms rising to a peak at moderate deprivation before declining again toward the highest deprivation decile. The remaining income quintiles showed comparatively flat or mildly declining trajectories.

In sparsely populated areas, trajectories across income quintiles were more heterogeneous, consistent with the weaker statistical evidence for this settlement type. The first and second income quintiles both increased with rising deprivation, the third quintile showed a moderate hump-shaped pattern, and the fourth and fifth quintiles remained comparatively flat. Figure 4 shows these patterns across all four settlement types and five income quintiles.

## Discussion

While the associations between household income, neighbourhood deprivation, and depressive symptoms are individually well established [35, 36], the central contribution of this study lies in demonstrating that these associations differ systematically across settlement structures. Neighbourhood deprivation showed a curvilinear association with depressive symptoms, and formal interaction tests confirmed that the shape of this association varied significantly by settlement type. In cities, this variation took the form of a clear buffering pattern, with the pronounced curvilinear association concentrated among the lowest income quintile; in other settlement types, income-related variation followed more complex, non-monotonic patterns.

### Interplay of individual and environmental influences

One of the salient findings of this study is the non-linear association between neighbourhood deprivation and depressive symptoms, consistent with ecological models of mental health [14, 15]. At the national level, however, income did not moderate this association in a straightforward, monotonic way. Only the third income quintile differed significantly from the lowest-income reference group, while the highest-income quintile, where a buffering effect would be expected to be most pronounced, did not differ significantly. This suggests that the relationship between income and neighbourhood-level vulnerability is more complex than a simple gradient of protection.

This pattern is consistent with transactional stress models [12], which propose that socioeconomic disadvantage and adverse contextual conditions compound one another. Where resources are scarce, residents may face heightened financial pressure, limited access to quality healthcare, and restricted social support, conditions that could jointly intensify mental distress. Individuals with greater financial resources may draw on greater autonomy, for instance through private transportation, better housing, or easier access to mental health services, which could help explain why the buffering pattern observed in this study was concentrated among the lowest income quintile in cities specifically, rather than reflecting a general nationwide gradient.

Our findings also confirmed well-documented sex differences, with women reporting more depressive symptoms, and identified individuals unable to work due to long-term health problems as a particularly vulnerable group. The lower symptom levels among older adults may partly reflect cohort-related vulnerability, given evidence of larger pandemic-related increases in depressive symptoms among younger cohorts in Germany [37–39], rather than a straightforward age effect.

### Urban–rural differences

Formal interaction tests confirmed substantial heterogeneity in how deprivation and household income relate to depressive symptoms across major cities, urban counties, rural counties, and sparsely populated rural areas. The most pronounced pattern emerged in cities, where higher GISD scores were associated with a marked increase in depressive symptoms among the lowest income group. The complex social fabric of metropolitan environments, marked by both pronounced deprivation in certain districts and pockets of affluence, may be associated with heightened psychosocial stress. Further, living in cities often entails crowded housing and greater anonymity. Healthcare providers, including mental health services, also tend to be unevenly distributed within cities, with lower provider density in more deprived districts despite high citywide averages [40], a pattern that may be linked to the mental toll of neighbourhood disadvantage observed in exactly those areas.

This pattern did not generalise across the remaining settlement types. Urban counties showed a markedly flatter association between deprivation and depressive symptoms than cities; this may reflect differences in the composition of these areas, residential patterns, or other contextual characteristics not measured here, and remains speculative pending future research using direct measures of social cohesion, housing conditions, healthcare accessibility, and local infrastructure. In rural and sparsely populated counties, the association did not merely weaken but reversed in direction, with predicted symptoms highest at moderate rather than extreme levels of deprivation. Together, these findings suggest that the pronounced, accelerating association between deprivation and depressive symptoms identified in this study is not a general feature of disadvantaged areas, but appears specifically associated with the dense, unequal conditions characteristic of major cities.

These findings should not be interpreted as evidence of a general “urban penalty”, but rather as indicating that socioeconomic deprivation is associated with depressive symptoms differently across settlement contexts, with a distinct, accelerating pattern emerging specifically in major cities.

### Implications for policy and practice

Given that both individual socioeconomic factors and neighbourhood contexts are significant correlates of depression, targeted interventions must address these dual domains. At the individual level, strategies to enhance employment opportunities, raise wages, and expand access to social welfare programs may diminish the vulnerability of low-income groups, particularly in areas of high deprivation. Policymakers could incentivise investment in underprivileged neighbourhoods through the development of affordable housing, improved public transportation, and community-based health centres.

On a broader scale, urban planning efforts may focus on addressing stark contrasts within city districts by introducing mixed-income housing projects, expanding green spaces, and ensuring equitable access to high-quality educational facilities. In rural settings, strengthening healthcare infrastructure, encouraging eHealth initiatives, such as telemedicine, online interventions, and enhancing social support networks could offset the limitations of geographic distance and lower population density. Importantly, tailoring these measures to regional specificities—whether major cities or sparsely populated areas— may contribute to more context-sensitive approaches to mental health promotion.

### Limitations and strengths

Limitations. First, the cross-sectional nature of our data constrains causal inference: we cannot conclusively determine whether neighbourhood deprivation leads to increased depressive symptoms or whether individuals with higher baseline distress selectively migrate to, or remain in, more deprived areas. Such residential self-selection may also be driven by additional unobserved characteristics, including personality traits, social resources, health status, or life circumstances, that were not captured in our data. Second, data protection regulations meant that county identifiers and geographic boundary information were not accessible beyond aggregated deprivation deciles and settlement-type classifications. This precluded the construction of spatial adjacency structures (e.g., contiguity-based weights) required for spatially explicit models such as spatial lag or spatial error regression, and may have obscured additional spatial trends in the association between deprivation and depressive symptoms. Third, data collection spanned both 2019 and 2020, thereby covering periods before and during the first COVID-19 lockdown in Germany. While our robustness checks indicated only modest differences between these periods, the pandemic constitutes a major contextual factor that may have influenced depressive symptom levels in ways not fully captured by our models. Future research should therefore examine pandemic-related mental health effects in more detail, ideally linking survey data to local infection rates, lockdown severity, and neighbourhood-level exposures.

Strengths. Despite these caveats, the study draws on a large, nationally representative dataset, bolstering external validity. The inclusion of diverse settlement structures—major cities, urban counties, rural counties, and sparsely populated rural areas— combined with formal interaction tests rather than descriptive comparisons alone, allowed for a rigorous examination of how regional characteristics intersect with individual socioeconomic conditions. Furthermore, the polynomial (non-linear) approach to modelling deprivation extends prior methodologies that primarily assumed linear effects, thereby offering a more refined understanding of how neighbourhood-level factors impact mental health.

### Directions for future research

Building on the insights offered by this cross-sectional investigation, future research efforts would benefit from longitudinal designs capable of illuminating the temporal sequence and causal pathways underlying socioeconomic and environmental determinants of mental health, complemented by multilevel modelling approaches that appropriately account for the nested structure of individuals within neighbourhoods. In addition, incorporating more granular environmental metrics, such as geospatial data on green space, noise pollution, or transport connectivity, alongside the deprivation, income, and settlement-level factors examined here, rather than in isolation, may help clarify which specific environmental features contribute to the patterns identified in this study. Further exploration of intersecting sociodemographic characteristics (e.g., age, sex, and migration background) and potential psychosocial mediators (such as coping strategies, perceived stress, and social support) is also warranted to capture a more complete picture of how social inequities compound or moderate neighbourhood effects. Finally, policy-focused evaluations, preferably in the form of longitudinal natural experiments or community-based interventions, are critical for assessing whether strategic enhancements to housing, social services, or urban infrastructure can tangibly reduce the burden of depressive symptoms, especially in highly deprived areas. Future research should also examine these processes in cross-national comparative designs to clarify how different healthcare and welfare systems may correspond to variation in neighbourhood effects, even under similar geospatial conditions. Moreover, given our evidence for non-linear deprivation effects, identifying threshold dynamics and testing non-linear models across diverse contexts represents an important next step. Through such combined approaches, future studies can strengthen the evidence base, inform targeted interventions, and ultimately contribute to a more equitable distribution of mental health resources.

## Conclusion

In summary, our results underscore the multifaceted relationship between individual socioeconomic factors and the broader environmental context in which people live. While higher neighbourhood deprivation is associated with the burden of depressive symptoms, individual resources, particularly higher income, are associated with lower symptom levels. These findings highlight the importance of both policy-level interventions aimed at reducing regional inequalities and targeted support for vulnerable individuals, tailored to the specific settlement contexts in which these associations were found to operate. Finally, an integrated approach, considering both personal and structural determinants of mental health, will be critical to alleviating the mental health burden associated with socioeconomic deprivation in Germany and beyond.

## Supplementary Information

Supplementary Material 1.

Supplementary Material 2.

## Data Availability

This study is based on data from the GEDA 2019/2020-EHIS survey provided by the Robert Koch Institute (RKI). The data were used under a data use agreement and are not publicly available. Access can be requested from the RKI subject to approval. Link: https://www.rki.de/DE/Aktuelles/Publikationen/Forschungsdaten/FDZ/Daten-nutzen/Scientific-Use-Files/scientific-use-files-node.html.
