# Supplementary material for: The mental cost of urban living: neighbourhood deprivation, income, and depressive symptoms in Germany
Source: BMC Public Health. 2026 Nov 14;26:3380. doi: 10.1186/s12889-026-30253-8 (PMC13769185; doi:10.1186/s12889-026-30253-8)
Supplement: Supplementary file 1 [file 12889_2026_30253_MOESM1_ESM.docx]

**Supplemental material to the manuscript**

The Mental Cost of Urban Living: A Study of Spatial Deprivation

**Supplement A. Settlement Structure**

**Figure A1.**

*German Settlement Structure Types of Districts (2020)*

**Note.** The map displays the spatial distribution of settlement structure types across Germany’s 401 districts as defined by the BBSR.

**Supplement B1. R packages used for the analyses**

Table A1 provides an overview of the R packages and versions used for the statistical analyses and visualization.

**Table B1.**

*R-Packages used for the Analysis*

| **Package** | **Version** | **Reference** |
| --- | --- | --- |
| car | 3.1-2 | Fox J, Weisberg S (2019). *An R Companion to Applied Regression*, Third Edition. Sage, Thousand Oaks CA. R package version 3.1-2, https://CRAN.R-project.org/package=car. |
| emmeans | 1.10.0 | Lenth RV (2024*). emmeans: Estimated Marginal Means, aka Least-Squares Means*. R package version 1.10.0, https://CRAN.R-project.org/package=emmeans. |
| foreign | 0.8-86 | R Core Team (2023). *foreign: Read Data Stored by 'Minitab', 'S', 'SAS', 'SPSS', 'Stata', 'Systat'.* R package version 0.8-86, https://CRAN.R-project.org/package=foreign. |
| ggplot2 | 3.5.1 | Wickham H (2016). *ggplot2: Elegant Graphics for Data Analysis*. Springer-Verlag New York. |
| ggpubr | 0.6.0 | Kassambara A (2023). *ggpubr: 'ggplot2' Based Publication Ready Plots*. R package version 0.6.0, https://CRAN.R-project.org/package=ggpubr. |
| ggsignif | 0.6.4 | Ahlmann-Eltze C, Patil I (2021). *ggsignif: R Package for Displaying Significance Brackets for 'ggplot2'*. Journal of Open Source Software, 6(60), 3266. doi:10.21105/joss.03266. |
| haven | 2.5.4 | Wickham H, Miller E, Smith D (2023). *haven: Import and Export 'SPSS', 'Stata' and 'SAS' Files*. R package version 2.5.4, https://CRAN.R-project.org/package=haven. |
| janitor | 2.2.0 | Firke S (2023). *janitor: Simple Tools for Examining and Cleaning Dirty Data*. R package version 2.2.0, https://CRAN.R-project.org/package=janitor. |
| labelled | 2.13.0 | Larmarange J (2024). *labelled: Manipulating Labelled Data*. R package version 2.13.0, https://CRAN.R-project.org/package=labelled. |
| lme4 | 1.1-35.2 | Bates D, Mächler M, Bolker B, Walker S (2015). *Fitting Linear Mixed-Effects Models Using lme4*. Journal of Statistical Software, 67(1), 1–48. doi:10.18637/jss.v067.i01. |
| patchwork | 1.2.0 | Pedersen TL (2024). *patchwork: The Composer of Plots*. R package version 1.2.0, https://CRAN.R-project.org/package=patchwork. |
| psych | 2.4.3 | Revelle W (2024). *psych: Procedures for Psychological, Psychometric, and Personality Research*. Northwestern University, Evanston, Illinois. R package version 2.4.3, https://CRAN.R-project.org/package=psych. |
| rstatix | 0.7.2 | Kassambara A (2023). *rstatix: Pipe-Friendly Framework for Basic Statistical Tests*. R package version 0.7.2, https://CRAN.R-project.org/package=rstatix. |
| sjlabelled | 1.2.0 | Lüdecke D (2024). *sjlabelled: Labelled Data Utility Functions*. R package version 1.2.0, https://CRAN.R-project.org/package=sjlabelled. |
| sjmisc | 2.8.10 | Lüdecke D (2018). *sjmisc: Data and Variable Transformation Functions*. Journal of Open Source Software, 3(26), 754. doi:10.21105/joss.00754. |
| sjPlot | 2.8.16 | Lüdecke D (2024). *sjPlot: Data Visualization for Statistics in Social Science*. R package version 2.8.16, https://CRAN.R-project.org/package=sjPlot. |
| sjstats | 0.19.0 | Lüdecke D (2024). *sjstats: Statistical Functions for Regression Models (Version 0.19.0)*. doi:10.5281/zenodo.1284472. |
| stats | 4.4.1 | R Core Team (2024). *stats: The R Stats Package*. R package version 4.4.1, https://CRAN.R-project.org/package=stats. |
| tidyverse | 2.0.0 | Wickham H, Averick M, Bryan J, Chang W, D’Agostino McGowan L, François R, Grolemund G, Hayes A, Henry L, Hester J, Kuhn M, Pedersen TL, Miller E, Bache SM, Müller K, Ooms J, Robinson D, Seidel DP, Spinu V, Takahashi K, Vaughan D, Wilke C, Woo K, Yutani H (2019). *Welcome to the tidyverse*. Journal of Open Source Software, 4(43), 1686. doi:10.21105/joss.01686. |

**Note.** Listed are all R packages, including version numbers and recommended references, that were used for data management, statistical analyses, and visualization in this study.

**Supplement B2. Polynomial Model Comparisons: Quadratic vs. Cubic Specifications**

Table A2 shows the comparison between the quadratic and cubic model specifications. Adding the cubic term did not yield a significant improvement in model fit (*F*(5, 20,313) = 0.68, *p* = .638), and the AIC slightly increased, indicating a worse fit relative to the quadratic model. We therefore retained the more parsimonious quadratic specification for all main analyses.

**Table B2.**

*Comparison of Quadratic and Cubic Polynomial Models Predicting Depressive Symptoms (PHQ-8)*

|  | ***Res.Df*** | ***RSS*** | ***Df*** | ***Sum of Sq.*** | ***F*** | ***p*** | ***AIC*** |
| --- | --- | --- | --- | --- | --- | --- | --- |
| Quadratic Model | 20318 | 219079 |  |  |  |  | **106163.1** |
| Cubic Model | 20313 | 219042 | 5 | 36.701 | 0.6807 | **0.638** | 106169.7 |

**Note.** Res.Df = residual degrees of freedom; RSS = residual sum of squares; Df = difference in degrees of freedom; Sum of Sq. = sum of squares; F = F statistic; p = significance level; AIC = Akaike Information Criterion. Lower AIC indicate better model fit.

**Supplement B3. Robustness Checks: Survey Timing (2019 vs. 2020)**

To examine whether the timing of data collection influenced results, we compared participants surveyed in 2019 and 2020. As shown in Table A2, income distributions differed significantly between the two years, with fewer respondents in the lowest income quintile and more in the highest quintile in 2020. Moreover, mean PHQ-8 scores were significantly lower in 2020 compared to 2019. These differences indicate a shift in sample characteristics and symptom levels across years. However, the overall associations between neighbourhood deprivation, household income, and settlement type remained stable, suggesting that the main findings are robust to period effects.

**Table B3.**

*Comparison of Sample Characteristics and Depressive Symptoms (PHQ-8) Between 2019 and 2020*

| **Year (*n*)** | **Q1 (%)** | **Q2 (%)** | **Q3 (%)** | **Q4 (%)** | **Q5 (%)** | **PHQ-8, *M* (*SD*)** |
| --- | --- | --- | --- | --- | --- | --- |
| 2019 (10,892) | **12.9 %** | 18.6 % | 17.1 % | 23.1 % | 28.3 % | 3.15 (3.54) |
| 2020 (9,881) | 10.8 % | 17.0 % | 17.0 % | 23.7 % | **31.5 %** | 2.82 (3.37) |

**Note.** Q1–Q5 = income quintiles, where Q1 represents the lowest and Q5 the highest household income group. Income distributions differed significantly between 2019 and 2020, with fewer participants in Q1 and more in Q5 in 2020, χ²(4) = 46.14, p < .001. Mean PHQ-8 scores were also significantly lower in 2020 compared to 2019 (Δ*M* = –0.43, 95% *CI* [–0.53, –0.34], p < .001).

**Supplement C. Correlation matrices stratified by district structure**

**Table C1**

*Correlation Matrix of the key variables (cities)*

|  | *1.* | *2.* | *3.* | *4.* | *5.* |
| --- | --- | --- | --- | --- | --- |
| *1. PHQ-8* |  |  |  |  |  |
| *2. Gender* | .102*** |  |  |  |  |
| *3. Age* | -.045*** | .086*** |  |  |  |
| *4. Migration Status* | -.050*** | .005 | .175*** |  |  |
| *5. Income Quintiles* | -.184*** | -.091*** | .042*** | .098*** |  |
| *6. GISD* | .019 | -.006 | .086*** | .064*** | -.083*** |

**Note.** Correlations were computed using Pearson’s r with pairwise deletion. p < .05 = *, p < .01 = **, p < .001 = ***. PHQ-8 = Patient Health Questionnaire-8; GISD = German Index of Socioeconomic Deprivation.

**Table C2**

*Correlation Matrix of the key variables (urban)*

|  | *1.* | *2.* | *3.* | *4.* | *5.* |
| --- | --- | --- | --- | --- | --- |
| *1. PHQ-8* |  |  |  |  |  |
| *2. Gender* | .117*** |  |  |  |  |
| *3. Age* | -.055*** | .035** |  |  |  |
| *4. Migration Status* | -.065*** | .027* | .133*** |  |  |
| *5. Income Quintiles* | -.172*** | -.119*** | .028* | .109*** |  |
| *6. GISD* | .033** | .013 | .082*** | .043*** | -.102*** |

**Note.** Correlations were computed using Pearson’s r with pairwise deletion. p < .05 = *, p < .01 = **, p < .001 = ***. PHQ-8 = Patient Health Questionnaire-8; GISD = German Index of Socioeconomic Deprivation.

**Table C3**

*Correlation Matrix of the key variables (rural)*

|  | *1.* | *2.* | *3.* | *4.* | *5.* |
| --- | --- | --- | --- | --- | --- |
| *1. PHQ-8* |  |  |  |  |  |
| *2. Gender* | .117*** |  |  |  |  |
| *3. Age* | -.051** | .087*** |  |  |  |
| *4. Migration Status* | .005 | -.001 | .083*** |  |  |
| *5. Income Quintiles* | -.189*** | -.123*** | .001 | .033 |  |
| *6. GISD* | -.003 | -.010 | .099*** | .096*** | -.103*** |

**Note.** Correlations were computed using Pearson’s r with pairwise deletion. p < .05 = *, p < .01 = **, p < .001 = ***. PHQ-8 = Patient Health Questionnaire-8; GISD = German Index of Socioeconomic Deprivation.

**Table C4**

*Correlation Matrix of the key variables (sparsely)*

|  | *1.* | *2.* | *3.* | *4.* | *5.* |
| --- | --- | --- | --- | --- | --- |
| *1. PHQ-8* |  |  |  |  |  |
| *2. Gender* | .080*** |  |  |  |  |
| *3. Age* | -.027 | .045* |  |  |  |
| *4. Migration Status* | -.031 | -.014 | .080*** |  |  |
| *5. Income Quintiles* | -.178*** | -.081*** | -.012 | .036 |  |
| *6. GISD* | .052** | -.025 | .096*** | .089*** | -.086*** |

**Note.** Correlations were computed using Pearson’s r with pairwise deletion. p < .05 = *, p < .01 = **, p < .001 = ***. PHQ-8 = Patient Health Questionnaire-8; GISD = German Index of Socioeconomic Deprivation.

**Supplement D. Complete Regression Tables**

**Table D1.**

*Weighted Regression Model Predicting Depressive Symptoms (PHQ-8) – Model 1*

|  | **PHQ-8** | | |
| --- | --- | --- | --- |
| *Predictors* | *Beta* | *CI* | *p* |
| Intercept | .71 | .58 – .84 | **<.001** |
| Female | .16 | .13 – .19 | **<.001** |
| Age | -.07 | -.09 – -.05 | **<.001** |
| Household Type [Single parent with child] | -.12 | -.20 – -.05 | **.001** |
| Household Type [Couple no children] | -.14 | -.24 – -.04 | **.007** |
| Household Type [Couple with children] | -.24 | -.35 – -.14 | **<.001** |
| Household Type [Other type of household] | -.18 | -.23 – -.12 | **<.001** |
| Living without partner | .02 | -.08 – .12 | .671 |
| Employment [Not employed for other reasons] | .03 | -.10 – .16 | .667 |
| Employment [Retired] | -.46 | -.54 – -.37 | **<.001** |
| Employment [Unable to work due to long-term health problems] | .89 | .79 – .99 | **<.001** |
| Employment [Employed] | -.46 | -.54 – -.38 | **<.001** |
| Employment [Student] | -.43 | -.52 – -.34 | **<.001** |
| Employment [Homemaker] | -.44 | -.55 – -.33 | **<.001** |
| Employment [Military or Civil Service] | -.21 | -.63 – .21 | .326 |
| Without Migration Background | -.16 | -.20 – -.12 | **<.001** |
| Household Equivalent Income [2^nd^ quint.] | -.11 | -.17 – -.05 | **<.001** |
| Household Equivalent Income [3^rd^ quint.] | -.16 | -.22 – -.09 | **<.001** |
| Household Equivalent Income [4^th^ quint.] | -.27 | -.33 – -.21 | **<.001** |
| Household Equivalent Income [5^th^ quint.] | -.35 | -.41 – -.29 | **<.001** |
| Deprivation | -.01 | -.04 – .02 | .469 |
| Deprivation [quadratic] | .04 | .01 – .07 | **.011** |
| Household Equivalent Income [2] × Deprivation | .03 | -.01 – .07 | .198 |
| Household Equivalent Income [3] × Deprivation | .04 | -.00 – .08 | .081 |
| Household Equivalent Income [4] × Deprivation | .01 | -.04 – .05 | .802 |
| Household Equivalent Income [5] × Deprivation | -.00 | -.05 – .04 | .826 |
| Household Equivalent Income [2] × Deprivation [quadratic] | -.03 | -.07 – .01 | .146 |
| Household Equivalent Income [3] × Deprivation [quadratic] | -.06 | -.10 – -.01 | **.014** |
| Household Equivalent Income [4] × Deprivation [quadratic] | -.03 | -.07 – .01 | .179 |
| Household Equivalent Income [5] × Deprivation [quadratic] | -.02 | -.06 – .02 | .392 |
| Observations | 20,348 | | |
| *R^2^* / *R^2^* adjusted | .128 / .127 | | |

**Note.** Reference Group for Female is Male; Reference Group Household Type is Single Person Household; Reference for Living without Partner is Living with Partner; Reference for Employment status is Unemployed; Reference for Household Equivalent Income is under 1^st^ quintile; Reference for Without Migration Background is With Migration Background.

**Table D2.**

*Weighted Regression Models Predicting Depressive Symptoms (PHQ-8), Stratified by Settlement Type – Model 2 to 5*

|  | **Cities** | | | | **Urban** | | | **Rural** | | | | **Sparsely Populated** | | |
| --- | --- | --- | --- | --- | --- | --- | --- | --- | --- | --- | --- | --- | --- | --- |
| *Predictors* | | *Beta* | *CI* | *p* | *Beta* | *CI* | *p* | | *Beta* | *CI* | *p* | *Beta* | *CI* | *p* |
| Intercept | | .22 | -.02 – .45 | .075 | 1.14 | .93 – 1.36 | **<.001** | | .93 | .56 – 1.30 | **<.001** | .62 | .23 – 1.01 | **.002** |
| Female | | .19 | .15 – .24 | **<.001** | .17 | .13 – .21 | **<.001** | | .14 | .07 – .21 | **<.001** | .09 | .02 – .17 | **.016** |
| Age | | -.08 | -.12 – -.04 | **<.001** | -.05 | -.09 – -.02 | **.003** | | -.07 | -.13 – -.01 | **.023** | -.11 | -.17 – -.05 | **<.001** |
| Household Type [Single parent with child] | | -.15 | -.28 – -.02 | **.023** | -.15 | -.27 – -.03 | **.015** | | -.21 | -.41 – -.00 | **.046** | .02 | -.18 – .22 | .876 |
| Household Type [Couple no children] | | -.12 | -.33 – .08 | .245 | -.15 | -.32 – .02 | .078 | | -.31 | -.57 – -.06 | **.014** | -.04 | -.31 – .22 | .750 |
| Household Type [Couple with children] | | -.19 | -.40 – .02 | .069 | -.32 | -.49 – -.15 | **<.001** | | -.42 | -.68 – -.16 | **.001** | -.07 | -.34 – .20 | .609 |
| Household Type [Other type of household] | | -.12 | -.20 – -.03 | **.010** | -.17 | -.26 – -.08 | **<.001** | | -.29 | -.44 – -.15 | **<.001** | -.21 | -.39 – -.04 | **.015** |
| Without Migration Background | | -.16 | -.22 – -.10 | **<.001** | -.23 | -.30 – -.17 | **<.001** | | .00 | -.15 – .15 | .981 | .04 | -.10 – .18 | .574 |
| Living without partner | | .05 | -.15 – .25 | .617 | -.04 | -.20 – .12 | .645 | | -.09 | -.32 – .15 | .476 | .12 | -.13 – .36 | .362 |
| Employment [Not employed for other reasons] | | .37 | .17 – .57 | **<.001** | -.41 | -.65 – -.18 | **.001** | | .05 | -.30 – .40 | .790 | -.16 | -.60 – .29 | .495 |
| Employment [Retired] | | -.17 | -.30 – -.03 | **.015** | -.71 | -.85 – -.57 | **<.001** | | -.54 | -.79 – -.29 | **<.001** | -.51 | -.76 – -.26 | **<.001** |
| Employment [Unable to work due to long-term health problems] | | .82 | .66 – .98 | **<.001** | 1.02 | .84 – 1.19 | **<.001** | | .75 | .46 – 1.05 | **<.001** | .89 | .59 – 1.19 | **<.001** |
| Employment [Employed] | | -.25 | -.36 – -.13 | **<.001** | -.64 | -.77 – -.51 | **<.001** | | -.49 | -.73 – -.25 | **<.001** | -.63 | -.87 – -.39 | **<.001** |
| Employment [Student] | | -.30 | -.43 – -.16 | **<.001** | -.62 | -.79 – -.46 | **<.001** | | -.28 | -.58 – .01 | .062 | -.39 | -.74 – -.05 | **.025** |
| Employment [Homemaker] | | -.35 | -.54 – -.16 | **<.001** | -.51 | -.67 – -.34 | **<.001** | | -.55 | -.88 – -.22 | **.001** | -.78 | -1.14 – -.42 | **<.001** |
| Employment [Military or Civil Service] | | -.51 | -1.42 – .40 | .275 | .22 | -.46 – .89 | .524 | | -1.27 | -2.74 – .21 | .093 | -.26 | -1.07 – .55 | .530 |
| Household Equivalent Income [2^nd^ quint.] | | .03 | -.07 – .14 | .533 | -.22 | -.31 – -.13 | **<.001** | | -.24 | -.39 – -.09 | **.002** | -.09 | -.27 – .09 | .345 |
| Household Equivalent Income [3^rd^ quint.] | | .05 | -.07 – .17 | .393 | -.30 | -.40 – -.21 | **<.001** | | -.27 | -.44 – -.11 | **.001** | -.11 | -.30 – .08 | .270 |
| Household Equivalent Income [4^th^ quint.] | | -.12 | -.23 – -.01 | **.040** | -.37 | -.46 – -.28 | **<.001** | | -.43 | -.59 – -.27 | **<.001** | -.23 | -.42 – -.04 | **.016** |
| Household Equivalent Income [5^th^ quint.] | | -.14 | -.25 – -.02 | **.018** | -.47 | -.56 – -.38 | **<.001** | | -.48 | -.66 – -.31 | **<.001** | -.38 | -.58 – -.18 | **<.001** |
| Deprivation | | -.01 | -.06 – .04 | .650 | -.09 | -.14 – -.04 | **.001** | | .04 | -.04 – .12 | .355 | -.02 | -.11 – .08 | .756 |
| Deprivation [quadratic] | | .24 | .18 – .30 | **<.001** | -.03 | -.07 – .02 | .221 | | -.13 | -.21 – -.05 | **.002** | -.06 | -.17 – .05 | .271 |
| Household Equivalent Income [2] × Deprivation | | -.03 | -.10 – .04 | .417 | .14 | .07 – .21 | **<.001** | | -.11 | -.22 – .00 | .056 | .10 | -.02 – .23 | .103 |
| Household Equivalent Income [3] × Deprivation | | .07 | -.01 – .15 | .086 | .13 | .05 – .20 | **.001** | | -.10 | -.21 – .02 | .105 | -.02 | -.16 – .11 | .726 |
| Household Equivalent Income [4] × Deprivation | | -.01 | -.09 – .06 | .737 | .10 | .03 – .17 | **.006** | | -.08 | -.19 – .04 | .175 | -.02 | -.16 – .12 | .787 |
| Household Equivalent Income [5] × Deprivation | | .01 | -.06 – .08 | .830 | .07 | .00 – .14 | **.046** | | -.08 | -.19 – .04 | .201 | -.01 | -.16 – .14 | .899 |
| Household Equivalent Income [2] × Deprivation [quadratic] | | -.21 | -.29 – -.12 | **<.001** | .05 | -.01 – .11 | .111 | | .11 | -.00 – .22 | .055 | .02 | -.12 – .16 | .801 |
| Household Equivalent Income [3] × Deprivation [quadratic] | | -.23 | -.33 – -.14 | **<.001** | .02 | -.05 – .08 | .599 | | .10 | -.02 – .21 | .092 | -.05 | -.19 – .09 | .499 |
| Household Equivalent Income [4] × Deprivation [quadratic] | | -.19 | -.28 – -.11 | **<.001** | .02 | -.04 – .08 | .481 | | .11 | -.00 – .22 | .053 | .06 | -.08 – .20 | .416 |
| Household Equivalent Income [5] × Deprivation [quadratic] | | -.21 | -.29 – -.12 | **<.001** | .05 | -.01 – .11 | .086 | | .13 | .01 – .24 | **.029** | .01 | -.14 – .15 | .922 |
| Observations | 7,224 | | | | 7,999 | | | | 2,690 | | | 2,435 | | |
| *R*^2^ / *R*^2^ adjusted | .120 / .117 | | | | .155 / .152 | | | | .139 / .130 | | | .144 / .133 | | |

**Note.** Reference Group for Female is Male; Reference Group Household Type is Single Person Household; Reference for Living without Partner is Living with Partner; Reference for Employment status is Unemployed; Reference for Household Equivalent Income is under 1^st^ quintile; Reference for Without Migration Background is With Migration Background.
