# Supplementary material for: The mental cost of urban living: neighbourhood deprivation, income, and depressive symptoms in Germany
Source: BMC Public Health. 2026 Nov 14;26:3380. doi: 10.1186/s12889-026-30253-8 (PMC13769185; doi:10.1186/s12889-026-30253-8)
Supplement: Supplementary file 2 [file 12889_2026_30253_MOESM2_ESM.docx]

**Supplemental material to the manuscript**

The Mental Cost of Urban Living: A Study of Spatial Deprivation

**Supplement A. Settlement Structure**

Settlement type was classified according to the BBSR, based on three criteria: population share in large and medium-sized cities, population density, and population density excluding large and medium-sized cities.

- Urban counties: population share ≥50% in large/medium-sized cities combined with a density of ≥150 inhabitants/km², or a density excluding these cities of ≥150 inhabitants/km²
- Rural counties: population share ≥50% in large/medium-sized cities with a density below 150 inhabitants/km², or a population share <50% combined with a density excluding these cities of ≥100 inhabitants/km²
- Sparsely populated rural counties: population share <50% in large/medium-sized cities and a density excluding these cities below 100 inhabitants/km²

**Figure A1.**

*German Settlement Structure Types of Districts (2020)*

**Note.** The map displays the spatial distribution of settlement structure types across Germany’s 401 districts as defined by the BBSR.

**Supplement B1. R packages used for the analyses**

Table A1 provides an overview of the R packages and versions used for the statistical analyses and visualization.

**Table B1.**

*R-Packages used for the Analysis*

| **Package** | **Version** | **Reference** |
| --- | --- | --- |
| car | 3.1-2 | Fox J, Weisberg S (2019). *An R Companion to Applied Regression*, Third Edition. Sage, Thousand Oaks CA. R package version 3.1-2, https://CRAN.R-project.org/package=car. |
| emmeans | 1.10.0 | Lenth RV (2024*). emmeans: Estimated Marginal Means, aka Least-Squares Means*. R package version 1.10.0, https://CRAN.R-project.org/package=emmeans. |
| foreign | 0.8-86 | R Core Team (2023). *foreign: Read Data Stored by 'Minitab', 'S', 'SAS', 'SPSS', 'Stata', 'Systat'.* R package version 0.8-86, https://CRAN.R-project.org/package=foreign. |
| ggplot2 | 3.5.1 | Wickham H (2016). *ggplot2: Elegant Graphics for Data Analysis*. Springer-Verlag New York. |
| ggpubr | 0.6.0 | Kassambara A (2023). *ggpubr: 'ggplot2' Based Publication Ready Plots*. R package version 0.6.0, https://CRAN.R-project.org/package=ggpubr. |
| ggsignif | 0.6.4 | Ahlmann-Eltze C, Patil I (2021). *ggsignif: R Package for Displaying Significance Brackets for 'ggplot2'*. Journal of Open Source Software, 6(60), 3266. doi:10.21105/joss.03266. |
| haven | 2.5.4 | Wickham H, Miller E, Smith D (2023). *haven: Import and Export 'SPSS', 'Stata' and 'SAS' Files*. R package version 2.5.4, https://CRAN.R-project.org/package=haven. |
| janitor | 2.2.0 | Firke S (2023). *janitor: Simple Tools for Examining and Cleaning Dirty Data*. R package version 2.2.0, https://CRAN.R-project.org/package=janitor. |
| labelled | 2.13.0 | Larmarange J (2024). *labelled: Manipulating Labelled Data*. R package version 2.13.0, https://CRAN.R-project.org/package=labelled. |
| lme4 | 1.1-35.2 | Bates D, Mächler M, Bolker B, Walker S (2015). *Fitting Linear Mixed-Effects Models Using lme4*. Journal of Statistical Software, 67(1), 1–48. doi:10.18637/jss.v067.i01. |
| patchwork | 1.2.0 | Pedersen TL (2024). *patchwork: The Composer of Plots*. R package version 1.2.0, https://CRAN.R-project.org/package=patchwork. |
| psych | 2.4.3 | Revelle W (2024). *psych: Procedures for Psychological, Psychometric, and Personality Research*. Northwestern University, Evanston, Illinois. R package version 2.4.3, https://CRAN.R-project.org/package=psych. |
| rstatix | 0.7.2 | Kassambara A (2023). *rstatix: Pipe-Friendly Framework for Basic Statistical Tests*. R package version 0.7.2, https://CRAN.R-project.org/package=rstatix. |
| sjlabelled | 1.2.0 | Lüdecke D (2024). *sjlabelled: Labelled Data Utility Functions*. R package version 1.2.0, https://CRAN.R-project.org/package=sjlabelled. |
| sjmisc | 2.8.10 | Lüdecke D (2018). *sjmisc: Data and Variable Transformation Functions*. Journal of Open Source Software, 3(26), 754. doi:10.21105/joss.00754. |
| sjPlot | 2.8.16 | Lüdecke D (2024). *sjPlot: Data Visualization for Statistics in Social Science*. R package version 2.8.16, https://CRAN.R-project.org/package=sjPlot. |
| sjstats | 0.19.0 | Lüdecke D (2024). *sjstats: Statistical Functions for Regression Models (Version 0.19.0)*. doi:10.5281/zenodo.1284472. |
| stats | 4.4.1 | R Core Team (2024). *stats: The R Stats Package*. R package version 4.4.1, https://CRAN.R-project.org/package=stats. |
| tidyverse | 2.0.0 | Wickham H, Averick M, Bryan J, Chang W, D’Agostino McGowan L, François R, Grolemund G, Hayes A, Henry L, Hester J, Kuhn M, Pedersen TL, Miller E, Bache SM, Müller K, Ooms J, Robinson D, Seidel DP, Spinu V, Takahashi K, Vaughan D, Wilke C, Woo K, Yutani H (2019). *Welcome to the tidyverse*. Journal of Open Source Software, 4(43), 1686. doi:10.21105/joss.01686. |

**Note.** Listed are all R packages, including version numbers and recommended references, that were used for data management, statistical analyses, and visualization in this study.

**Supplement Figure B2.**

*Comparison of LOESS and OLS Trend Estimates*

**Note.** Comparison of nonparametric LOESS smoothing and ordinary least squares (OLS) regression in modelling the association between neighbourhood socioeconomic deprivation (GISD) and depressive symptoms (PHQ-8). Panel A shows the full GISD range (deciles 1–10), while Panel B illustrates the relationship across household equivalent income quintiles. Shaded areas represent 95% confidence intervals.

**Supplement B2. Polynomial Model Comparisons: Quadratic vs. Cubic Specifications**

Table B2 shows the comparison between the quadratic and cubic model specifications. Adding the cubic term did not yield a significant improvement in model fit (*F*(5, 20,313) = 0.68, *p* = .638), and the AIC slightly increased, indicating a worse fit relative to the quadratic model. We therefore retained the more parsimonious quadratic specification for all main analyses.

**Table B2.**

*Comparison of Quadratic and Cubic Polynomial Models Predicting Depressive Symptoms (PHQ-8)*

|  | ***Res.Df*** | ***RSS*** | ***Df*** | ***Sum of Sq.*** | ***F*** | ***p*** | ***AIC*** |
| --- | --- | --- | --- | --- | --- | --- | --- |
| Quadratic Model | 20318 | 219079 |  |  |  |  | **106163.1** |
| Cubic Model | 20313 | 219042 | 5 | 36.701 | 0.6807 | **0.638** | 106169.7 |

**Note.** Res.Df = residual degrees of freedom; RSS = residual sum of squares; Df = difference in degrees of freedom; Sum of Sq. = sum of squares; F = F statistic; p = significance level; AIC = Akaike Information Criterion. Lower AIC indicate better model fit.

**Supplement B3. Robustness Checks: Survey Timing (2019 vs. 2020)**

To examine whether the timing of data collection influenced results, we compared participants surveyed in 2019 and 2020. As shown in Table B3, income distributions differed significantly between the two years, with fewer respondents in the lowest income quintile and more in the highest quintile in 2020. Moreover, mean PHQ-8 scores were significantly lower in 2020 compared to 2019. These differences indicate a shift in sample characteristics and symptom levels across years. However, the overall associations between neighbourhood deprivation, household income, and settlement type remained stable, suggesting that the main findings are robust to period effects.

**Table B3.**

*Comparison of Sample Characteristics and Depressive Symptoms (PHQ-8) Between 2019 and 2020*

| **Year (*n*)** | **Q1 (%)** | **Q2 (%)** | **Q3 (%)** | **Q4 (%)** | **Q5 (%)** | **PHQ-8, *M* (*SD*)** |
| --- | --- | --- | --- | --- | --- | --- |
| 2019 (10,892) | **12.9 %** | 18.6 % | 17.1 % | 23.1 % | 28.3 % | 3.15 (3.54) |
| 2020 (9,881) | 10.8 % | 17.0 % | 17.0 % | 23.7 % | **31.5 %** | 2.82 (3.37) |

**Note.** Q1–Q5 = income quintiles, where Q1 represents the lowest and Q5 the highest household income group. Income distributions differed significantly between 2019 and 2020, with fewer participants in Q1 and more in Q5 in 2020, χ²(4) = 46.14, p < .001. Mean PHQ-8 scores were also significantly lower in 2020 compared to 2019 (Δ*M* = –0.43, 95% *CI* [–0.53, –0.34], p < .001).

**Supplement B4. Variance Inflation Factors for Model 1**

| **Predictor** | **GVIF** | **Df** | **GVIF^(1/(2·Df))** |
| --- | --- | --- | --- |
| Sex | 1.05 | 1 | 1.03 |
| Age | 2.93 | 1 | 1.71 |
| Household type | 20.55 | 4 | 1.46 |
| Living without partner | 14.33 | 1 | 3.79 |
| Employment | 4.05 | 7 | 1.11 |
| Migration background | 1.06 | 1 | 1.03 |
| Household equivalent income | 25.97 | **4** | **1.50** |
| Deprivation | 5.38 | 1 | 2.32 |
| Deprivation [quadratic] | 5.09 | 1 | 2.26 |
| Household equivalent income × Deprivation | 5.77 | **4** | **1.25** |
| Household equivalent income × Deprivation [quadratic] | 67.73 | **4** | **1.69** |

**Note.** GVIF = generalized variance inflation factor (Fox & Monette 1992). For predictors with more than one degree of freedom (Df), GVIF^1/(2·Df) is reported for comparability with the conventional √VIF threshold. Values below 2 are generally considered unproblematic.

Fox, J., & Monette, G. (1992). Generalized collinearity diagnostics. *Journal of the American Statistical Association*, *87*(417), 178-183.

**Supplement C. Correlation matrices stratified by district structure**

**Table C1**

*Correlation Matrix of the key variables (cities)*

|  | *1.* | *2.* | *3.* | *4.* | *5.* |
| --- | --- | --- | --- | --- | --- |
| *1. PHQ-8* |  |  |  |  |  |
| *2. Gender* | .102*** |  |  |  |  |
| *3. Age* | -.045*** | .086*** |  |  |  |
| *4. Migration Status* | -.050*** | .005 | .175*** |  |  |
| *5. Income Quintiles* | -.184*** | -.091*** | .042*** | .098*** |  |
| *6. GISD* | .019 | -.006 | .086*** | .064*** | -.083*** |

**Note.** Correlations were computed using Pearson’s r with pairwise deletion. p < .05 = *, p < .01 = **, p < .001 = ***. PHQ-8 = Patient Health Questionnaire-8; GISD = German Index of Socioeconomic Deprivation.

**Table C2**

*Correlation Matrix of the key variables (urban)*

|  | *1.* | *2.* | *3.* | *4.* | *5.* |
| --- | --- | --- | --- | --- | --- |
| *1. PHQ-8* |  |  |  |  |  |
| *2. Gender* | .117*** |  |  |  |  |
| *3. Age* | -.055*** | .035** |  |  |  |
| *4. Migration Status* | -.065*** | .027* | .133*** |  |  |
| *5. Income Quintiles* | -.172*** | -.119*** | .028* | .109*** |  |
| *6. GISD* | .033** | .013 | .082*** | .043*** | -.102*** |

**Note.** Correlations were computed using Pearson’s r with pairwise deletion. p < .05 = *, p < .01 = **, p < .001 = ***. PHQ-8 = Patient Health Questionnaire-8; GISD = German Index of Socioeconomic Deprivation.

**Table C3**

*Correlation Matrix of the key variables (rural)*

|  | *1.* | *2.* | *3.* | *4.* | *5.* |
| --- | --- | --- | --- | --- | --- |
| *1. PHQ-8* |  |  |  |  |  |
| *2. Gender* | .117*** |  |  |  |  |
| *3. Age* | -.051** | .087*** |  |  |  |
| *4. Migration Status* | .005 | -.001 | .083*** |  |  |
| *5. Income Quintiles* | -.189*** | -.123*** | .001 | .033 |  |
| *6. GISD* | -.003 | -.010 | .099*** | .096*** | -.103*** |

**Note.** Correlations were computed using Pearson’s r with pairwise deletion. p < .05 = *, p < .01 = **, p < .001 = ***. PHQ-8 = Patient Health Questionnaire-8; GISD = German Index of Socioeconomic Deprivation.

**Table C4**

*Correlation Matrix of the key variables (sparsely)*

|  | *1.* | *2.* | *3.* | *4.* | *5.* |
| --- | --- | --- | --- | --- | --- |
| *1. PHQ-8* |  |  |  |  |  |
| *2. Gender* | .080*** |  |  |  |  |
| *3. Age* | -.027 | .045* |  |  |  |
| *4. Migration Status* | -.031 | -.014 | .080*** |  |  |
| *5. Income Quintiles* | -.178*** | -.081*** | -.012 | .036 |  |
| *6. GISD* | .052** | -.025 | .096*** | .089*** | -.086*** |

**Note.** Correlations were computed using Pearson’s r with pairwise deletion. p < .05 = *, p < .01 = **, p < .001 = ***. PHQ-8 = Patient Health Questionnaire-8; GISD = German Index of Socioeconomic Deprivation.

**Supplement D. Complete Regression Tables**

**Table D1.**

*Weighted Regression Model Testing the Moderating Role of Settlement Type (H4), Full Model*

|  | **PHQ-8** | | |
| --- | --- | --- | --- |
| *Predictors* | *Estimates* | *CI* | *p* |
| Intercept | 6.85 | 6.27 – 7.42 | **<.001** |
| Female | .63 | .53 – .74 | **<.001** |
| Age | -.02 | -.02 – -.01 | **<.001** |
| Household Type [Single parent with child] | -.49 | -.78 – -.19 | **.001** |
| Household Type [Couple no children] | -.56 | -.96 – -.15 | **.007** |
| Household Type [Couple with children] | -.96 | -1.37 – -.55 | **<.001** |
| Household Type [Other type of household] | -.70 | -.92 – -.49 | **<.001** |
| Living without partner | .07 | -.32 – .45 | .723 |
| Employment [Not employed for other reasons] | .14 | -.39 – .67 | .608 |
| Employment [Retired] | -1.79 | -2.12 – -1.45 | **<.001** |
| Employment [Unable to work due to long-term health problems] | 3.52 | 3.12 – 3.92 | **<.001** |
| Employment [Employed] | -1.79 | -2.09 – -1.48 | **<.001** |
| Employment [Student] | -1.70 | -2.08 – -1.33 | **<.001** |
| Employment [Homemaker] | -1.73 | -2.17 – -1.30 | **<.001** |
| Employment [Military or Civil Service] | -.81 | -2.46 – .83 | .333 |
| Without Migration Background | -.59 | -.75 – -.44 | **<.001** |
| Household Equivalent Income [2nd quint.] | -.44 | -.67 – -.20 | **<.001** |
| Household Equivalent Income [3rd quint.] | -.63 | -.88 – -.38 | **<.001** |
| Household Equivalent Income [4th quint.] | -1.08 | -1.33 – -.84 | **<.001** |
| Household Equivalent Income [5th quint.] | -1.39 | -1.64 – -1.14 | **<.001** |
| Deprivation | .03 | -.02 – .07 | .257 |
| Deprivation [quadratic] | .05 | .03 – .06 | **<.001** |
| Settlement Type [Urban County] | .20 | .01 – .39 | **.042** |
| Settlement Type [Rural County] | .41 | .18 – .64 | **.001** |
| Settlement Type [Sparsely Populated Rural County] | .21 | -.04 – .47 | .104 |
| Household Equivalent Income [2] × Deprivation | .03 | -.03 – .09 | .287 |
| Household Equivalent Income [3] × Deprivation | .04 | -.02 – .10 | .177 |
| Household Equivalent Income [4] × Deprivation | .00 | -.06 – .05 | .906 |
| Household Equivalent Income [5] × Deprivation | -.01 | -.07 – .04 | .641 |
| Household Equivalent Income [2] × Deprivation [quadratic] | -.01 | -.04 – .01 | .205 |
| Household Equivalent Income [3] × Deprivation [quadratic] | -.03 | -.05 – -.00 | **.023** |
| Household Equivalent Income [4] × Deprivation [quadratic] | -.01 | -.04 – .01 | .191 |
| Household Equivalent Income [5] × Deprivation [quadratic] | -.01 | -.03 – .01 | .361 |
| Settlement Type [Urban County] × Deprivation | -.04 | -.08 – .01 | .132 |
| Settlement Type [Rural County] × Deprivation | -.07 | -.13 – -.01 | **.022** |
| Settlement Type [Sparsely Populated Rural County] × Deprivation | .06 | -.02 – .13 | .120 |
| Settlement Type [Urban County] × Deprivation [quadratic] | -.03 | -.05 – -.02 | **<.001** |
| Settlement Type [Rural County] × Deprivation [quadratic] | -.06 | -.08 – -.03 | **<.001** |
| Settlement Type [Sparsely Populated Rural County] × Deprivation [quadratic] | -.06 | -.08 – -.03 | **<.001** |
| Observations | 20,348 |  |  |
| R² / R² adjusted | .131 / .129 |  |  |

**Note.** Reference Group for Female is Male; Reference Group Household Type is Single Person Household; Reference for Living without Partner is Living with Partner; Reference for Employment status is Unemployed; Reference for Household Equivalent Income is under 1st quintile; Reference for Without Migration Background is With Migration Background; Reference for Settlement Type is Cities. Model compared to Model 1 via nested F-test, F(9, 20309) = 5.97, p < .001.

**Table D2.**

*Weighted Regression Model Testing the Moderating Role of Settlement Type and Income (H5), Full Model*

|  | **PHQ-8** | | |
| --- | --- | --- | --- |
| *Predictors* | *Estimates* | *CI* | *p* |
| Intercept | 6.15 | 5.53 – 6.78 | **<.001** |
| Female | .63 | .52 – .73 | **<.001** |
| Age | -.01 | -.02 – -.01 | **<.001** |
| Household Type [Single parent with child] | -.53 | -.82 – -.24 | **<.001** |
| Household Type [Couple no children] | -.55 | -.96 – -.15 | **.007** |
| Household Type [Couple with children] | -.95 | -1.36 – -.55 | **<.001** |
| Household Type [Other type of household] | -.72 | -.93 – -.50 | **<.001** |
| Living without partner | .08 | -.31 – .46 | .688 |
| Employment [Not employed for other reasons] | .12 | -.41 – .65 | .649 |
| Employment [Retired] | -1.79 | -2.12 – -1.45 | **<.001** |
| Employment [Unable to work due to long-term health problems] | 3.53 | 3.12 – 3.93 | **<.001** |
| Employment [Employed] | -1.78 | -2.09 – -1.47 | **<.001** |
| Employment [Student] | -1.66 | -2.04 – -1.29 | **<.001** |
| Employment [Homemaker] | -1.73 | -2.17 – -1.29 | **<.001** |
| Employment [Military or Civil Service] | -.63 | -2.28 – 1.02 | .454 |
| Without Migration Background | -.59 | -.75 – -.43 | **<.001** |
| Household Equivalent Income [2nd quint.] | .26 | -.20 – .72 | .265 |
| Household Equivalent Income [3rd quint.] | .40 | -.09 – .90 | .109 |
| Household Equivalent Income [4th quint.] | -.33 | -.81 – .15 | .179 |
| Household Equivalent Income [5th quint.] | -.42 | -.90 – .05 | .080 |
| Deprivation, Cities & 1st quintile [reference] | .11 | .04 – .17 | **.001** |
| Deprivation [quadratic], Cities & 1st quintile [reference] | .10 | .07 – .12 | **<.001** |
| Settlement Type [Urban County] | 1.15 | .72 – 1.57 | **<.001** |
| Settlement Type [Rural County] | 1.56 | 1.06 – 2.06 | **<.001** |
| Settlement Type [Sparsely Populated Rural County] | .77 | .19 – 1.36 | **.010** |
| Household Equivalent Income [2] × Deprivation | -.13 | -.22 – -.04 | **.005** |
| Household Equivalent Income [3] × Deprivation | -.03 | -.13 – .06 | .479 |
| Household Equivalent Income [4] × Deprivation | -.10 | -.19 – -.01 | **.031** |
| Household Equivalent Income [5] × Deprivation | -.10 | -.19 – -.00 | **.040** |
| Household Equivalent Income [2] × Deprivation [quadratic] | -.08 | -.12 – -.05 | **<.001** |
| Household Equivalent Income [3] × Deprivation [quadratic] | -.09 | -.13 – -.05 | **<.001** |
| Household Equivalent Income [4] × Deprivation [quadratic] | -.08 | -.11 – -.04 | **<.001** |
| Household Equivalent Income [5] × Deprivation [quadratic] | -.08 | -.12 – -.05 | **<.001** |
| Settlement Type [Urban County] × Deprivation | -.28 | -.38 – -.17 | **<.001** |
| Settlement Type [Rural County] × Deprivation | .04 | -.11 – .18 | .619 |
| Settlement Type [Sparsely Populated Rural County] × Deprivation | .01 | -.18 – .20 | .916 |
| Settlement Type [Urban County] × Deprivation [quadratic] | -.11 | -.16 – -.07 | **<.001** |
| Settlement Type [Rural County] × Deprivation [quadratic] | -.18 | -.23 – -.13 | **<.001** |
| Settlement Type [Sparsely Populated Rural County] × Deprivation [quadratic] | -.13 | -.19 – -.07 | **<.001** |
| Household Equivalent Income [2] × Settlement Type [Urban County] | -.94 | -1.52 – -.35 | **.002** |
| Household Equivalent Income [3] × Settlement Type [Urban County] | -1.50 | -2.13 – -.88 | **<.001** |
| Household Equivalent Income [4] × Settlement Type [Urban County] | -1.07 | -1.68 – -.46 | **.001** |
| Household Equivalent Income [5] × Settlement Type [Urban County] | -1.35 | -1.95 – -.75 | **<.001** |
| Household Equivalent Income [2] × Settlement Type [Rural County] | -1.27 | -1.96 – -.58 | **<.001** |
| Household Equivalent Income [3] × Settlement Type [Rural County] | -1.59 | -2.33 – -.86 | **<.001** |
| Household Equivalent Income [4] × Settlement Type [Rural County] | -1.46 | -2.18 – -.75 | **<.001** |
| Household Equivalent Income [5] × Settlement Type [Rural County] | -1.60 | -2.33 – -.86 | **<.001** |
| Household Equivalent Income [2] × Settlement Type [Sparsely Populated Rural County] | -.74 | -1.52 – .05 | .066 |
| Household Equivalent Income [3] × Settlement Type [Sparsely Populated Rural County] | -.83 | -1.66 – .00 | .051 |
| Household Equivalent Income [4] × Settlement Type [Sparsely Populated Rural County] | -.45 | -1.27 – .37 | .281 |
| Household Equivalent Income [5] × Settlement Type [Sparsely Populated Rural County] | -.99 | -1.84 – -.15 | **.021** |
| Household Equivalent Income [2] × Settlement Type [Urban County] × Deprivation | .41 | .27 – .56 | **<.001** |
| Household Equivalent Income [3] × Settlement Type [Urban County] × Deprivation | .24 | .09 – .40 | **.002** |
| Household Equivalent Income [4] × Settlement Type [Urban County] × Deprivation | .27 | .12 – .42 | **<.001** |
| Household Equivalent Income [5] × Settlement Type [Urban County] × Deprivation | .26 | .11 – .41 | **.001** |
| Household Equivalent Income [2] × Settlement Type [Rural County] × Deprivation | -.10 | -.30 – .09 | .290 |
| Household Equivalent Income [3] × Settlement Type [Rural County] × Deprivation | -.17 | -.37 – .03 | .097 |
| Household Equivalent Income [4] × Settlement Type [Rural County] × Deprivation | -.09 | -.28 – .10 | .353 |
| Household Equivalent Income [5] × Settlement Type [Rural County] × Deprivation | -.11 | -.30 – .08 | .267 |
| Household Equivalent Income [2] × Settlement Type [Sparsely Populated Rural County] × Deprivation | .24 | -.00 – .49 | .054 |
| Household Equivalent Income [3] × Settlement Type [Sparsely Populated Rural County] × Deprivation | .07 | -.19 – .32 | .602 |
| Household Equivalent Income [4] × Settlement Type [Sparsely Populated Rural County] × Deprivation | -.06 | -.30 – .18 | .617 |
| Household Equivalent Income [5] × Settlement Type [Sparsely Populated Rural County] × Deprivation | .03 | -.22 – .28 | .793 |
| Household Equivalent Income [2] × Settlement Type [Urban County] × Deprivation [quadratic] | .12 | .06 – .17 | **<.001** |
| Household Equivalent Income [3] × Settlement Type [Urban County] × Deprivation [quadratic] | .10 | .04 – .16 | **.001** |
| Household Equivalent Income [4] × Settlement Type [Urban County] × Deprivation [quadratic] | .09 | .03 – .15 | **.002** |
| Household Equivalent Income [5] × Settlement Type [Urban County] × Deprivation [quadratic] | .12 | .06 – .17 | **<.001** |
| Household Equivalent Income [2] × Settlement Type [Rural County] × Deprivation [quadratic] | .15 | .08 – .22 | **<.001** |
| Household Equivalent Income [3] × Settlement Type [Rural County] × Deprivation [quadratic] | .16 | .08 – .23 | **<.001** |
| Household Equivalent Income [4] × Settlement Type [Rural County] × Deprivation [quadratic] | .15 | .08 – .22 | **<.001** |
| Household Equivalent Income [5] × Settlement Type [Rural County] × Deprivation [quadratic] | .17 | .10 – .24 | **<.001** |
| Household Equivalent Income [2] × Settlement Type [Sparsely Populated Rural County] × Deprivation [quadratic] | .08 | .00 – .16 | **.047** |
| Household Equivalent Income [3] × Settlement Type [Sparsely Populated Rural County] × Deprivation [quadratic] | .06 | -.02 – .15 | .150 |
| Household Equivalent Income [4] × Settlement Type [Sparsely Populated Rural County] × Deprivation [quadratic] | .11 | .03 – .19 | **.009** |
| Household Equivalent Income [5] × Settlement Type [Sparsely Populated Rural County] × Deprivation [quadratic] | .08 | -.00 – .17 | .056 |
| Observations | 20,348 |  |  |
| R² / R² adjusted | .135 / .132 |  |  |

**Note.** Reference Group for Female is Male; Reference Group Household Type is Single Person Household; Reference for Living without Partner is Living with Partner; Reference for Employment status is Unemployed; Reference for Household Equivalent Income is 1st quintile; Reference for Without Migration Background is With Migration Background; Reference for Settlement Type is Cities. Deprivation and Deprivation [quadratic] main effects apply to the joint reference group (Cities, 1st income quintile). Model compared to H4 model via nested F-test, F(36, 20273) = 2.93, p < .001.
